# Updating the Phylodynamics of Yellow Fever Virus 2016–2019 Brazilian Outbreak With New 2018 and 2019 São Paulo Genomes

**DOI:** 10.3389/fmicb.2022.811318

**Published:** 2022-04-14

**Authors:** Ana Paula Moreira Salles, Ana Catharina de Seixas Santos Nastri, Yeh-Li Ho, Luciana Vilas Boas Casadio, Deyvid Emanuel Amgarten, Santiago Justo Arévalo, Michele Soares Gomes-Gouvea, Flair Jose Carrilho, Fernanda de Mello Malta, João Renato Rebello Pinho

**Affiliations:** ^1^Department of Gastroenterology (LIM07), Faculdade de Medicina, Universidade de São Paulo, São Paulo, Brazil; ^2^Clinical Laboratory of Hospital Israelita Albert Einstein, São Paulo, Brazil; ^3^Department of Infectious and Parasitic Diseases, Hospital das Clínicas, Faculdade de Medicina, Universidade de São Paulo, São Paulo, Brazil; ^4^Departamento de Bioquímica, Instituto de Química, Universidade de São Paulo, São Paulo, Brazil; ^5^Facultad de Ciencias Biológicas, Universidad Ricardo Palma, Lima, Peru; ^6^Division of Clinical Laboratories (LIM 03), Hospital das Clínicas, Faculdade de Medicina, Universidade de São Paulo, São Paulo, Brazil

**Keywords:** yellow fever virus, next generation sequencing, outbreak, São Paulo, vaccine coverage

## Abstract

The recent outbreak of yellow fever (YF) in São Paulo during 2016–2019 has been one of the most severe in the last decades, spreading to areas with low vaccine coverage. The aim of this study was to assess the genetic diversity of the yellow fever virus (YFV) from São Paulo 2016–2019 outbreak, integrating the available genomic data with new genomes from patients from the Hospital das Clínicas da Faculdade de Medicina da Universidade de São Paulo (HCFMUSP). Using phylodynamics, we proposed the existence of new IE subclades, described their sequence signatures, and determined their locations and time of origin. Plasma or urine samples from acute severe YF cases (*n* = 56) with polymerase chain reaction (PCR) positive to YFV were submitted to viral genome amplification using 12 sets of primers. Thirty-nine amplified genomes were subsequently sequenced using next-generation sequencing (NGS). These 39 sequences, together with all the complete genomes publicly available, were aligned and used to determine nucleotide/amino acids substitutions and perform phylogenetic and phylodynamic analysis. All YFV genomes generated in this study belonged to the genotype South American I subgroup E. Twenty-one non-synonymous substitutions were identified among the new generated genomes. We analyzed two major clades of the genotypes IE, IE1, and IE2 and proposed the existence of subclades based on their sequence signatures. Also, we described the location and time of origin of these subclades. Overall, our findings provide an overview of YFV genomic characterization and phylodynamics of the 2016–2019 outbreak contributing to future virological and epidemiological studies.

## Introduction

Yellow fever (YF) is a tropical short-term disease transmitted by the bite of infected female mosquitoes. It has a large spectrum of symptoms, from an asymptomatic form to a severe and deadly hemorrhagic fever in humans and non-human primates (NHPs) (Beasley et al., 2015; Tabachnick, 2016). It is estimated to cause approximately 30,000 deaths out of 200,000 infections worldwide, mostly in Africa (Monath and Vasconcelos, 2015).

Yellow fever virus (YFV) belonging to the *Flaviviridae* family is the etiological agent of the YF. It is a positive-sense single-stranded RNA virus with a genome of approximately 11 kb that consists of a 5’ untranslated region (UTR), followed by a single open reading frame (ORF), and a 3’UTR (Chambers et al., 1990; Gómez et al., 2018). The ORF is further divided into three structural proteins (C, prM/M, and E) and seven non-structural proteins (NS1, NS2A, NS2B, NS3, NS4A, NS4B, and NS5).

Yellow fever is found mostly in Africa and the Americas with recurrent epidemics from the seventeenth century until the beginning of the twentieth century in Europe and North America. Currently, almost all the infections are from the endemic areas of Africa, and Central and South America.

Seven lineages of YFV have been identified so far: five in Africa (West Africa I and II, East Africa, East/Central Africa, and Angola) with an estimated genetic variance at the nucleotide level ranging between 10 and 23% (Mutebi et al., 2001; von Lindern et al., 2006) and two in Central and South America (South America I and II) with an estimated genetic diversity at the nucleotide level of 7% (Mutebi et al., 2001; Mir et al., 2017).

The South American I is the most prevalent genotype in Brazil, and it is divided into subgroups IA to IE (de Souza et al., 2010; Nunes et al., 2012). Only subgroups ID and IE have been detected circulating in Brazil, but since 2008, only subgroup IE has been detected. Recent studies speculated that the YFV strain, associated with the recent outbreaks (genotype IE), would have originated in the central–west region and then probably reached the southeast region (Cunha et al., 2019a; Delatorre et al., 2019).

YFV has been sporadically detected in non-human primates (NHPs) and human populations from enzootic and endemic areas from northern and central–western regions of Brazil before the twentieth century. However, during the last two decades, YFV has spread to the southeast region, reaching the Atlantic rainforest. After the end of 2016, an important increase in human cases have been reported in the south and southeast of Brazil (Silva et al., 2020).

According to the last Brazilian epidemiological report from the Ministry of Health, the metropolitan area of São Paulo City had 538 confirmed human cases and 184 deaths (34.2%) in 2018 and 66 confirmed human cases and 12 deaths (18.2%) during 2019 (Governo do Estado de São Paulo, 2019).

The aim of this study was to assess the genetic diversity and phylodynamics of YFV from the 2016–2019 outbreak, integrating the available genomic data with new genomes from patients from the Hospital das Clínicas da Faculdade de Medicina da Universidade de São Paulo (HCFMUSP). Using phylodynamics, we proposed the existence of new subclades, described their sequence signatures, and determined their locations and time of origin. This study may help in the surveillance, epidemiology studies, and to increase our understanding of the genetic diversity and spread of YFV.

## Materials and Methods

### Patients and Samples

Among 192 suspected YF cases followed at HCFMUSP during the 2018 and 2019 outbreaks, 56 patients that had their YF infection confirmed by qPCR were enrolled in this study. Blood and urine samples were collected at hospital admission. The methodology applied for detection and quantification of YFV-RNA in serum and urine samples was the same as that described by Casadio et al. (2019), noticing that all of them were tested for both wild and vaccine YFV strains, using specific primers and probes for each one of them. After that, we chose the earliest positive available sample of each patient to perform the viral genome sequencing.

We also performed a geopositioning analysis using the previously collected information and available data on patient residence and year of infection and mapped them using Google Maps^®^ (Google, 2021) tools available at the Google platform.

This study was conducted in compliance with the institutional guidelines, approved by the Ethical Committee from the Hospital das Clínicas da Faculdade de Medicina da Universidade de São Paulo (CEP/HCFMUSP; CAAE: 74535417.3,1001.0068), and all individuals signed written informed consent forms.

### Amplification and Sequencing of Yellow Fever Virus Genome

Viral RNA was isolated from 140 μl of serum or urine using QIAamp^®^ Viral RNA Mini Kit (Qiagen™, Hilden, Germany), according to the manufacturer protocol. After extraction, the RNA was reverse transcribed to cDNA using random primers and M-MLV Reverse Transcriptase 200 U/μl (Invitrogen™, Thermo Fisher Scientific Brand, Carlsbad, CA, United States), according to the manufacturer’s instructions. The cDNA was amplified by PCR using 12 different pairs of primers generating 11 overlapping PCR fragments covering the YFV genome (each fragment was amplified separately, 11 PCR reactions by sample; Supplementary Table 1).

The singleplex PCR reactions contained 35 μl of RNase-free H_2_O, 5 μl 10 × buffer, 1 μl of dNTP mix (10 nM), 1.5 μl of MgCl_2_ (50 nM), 1 μl of a set of primers (20 nM), 5 U of Platinum Taq DNA Polymerase (Invitrogen™, Thermo Fisher Scientific Brand, Carlsbad, CA, United States), and 5 μl of cDNA. The cycling protocol was initial denaturation at 94°C for 5 min, then 45 cycles of denaturation at 94°C for 30 s, annealing at 65°C for 30 s, and extension at 72°C for 90 s, followed by 72°C for 10 min, and 10°C up to the next step.

Among the 56 samples, we were able to amplify all overlapping PCR fragments from 40 YFV-positive samples (mean of Ct value = 26.7), at least one PCR fragment from the other 12 samples (mean of Ct value = 29.2), and we were not able to amplify any fragment in 4 samples (mean of Ct value = 30.8). The most difficult YFV genome region to amplify was the 3’UTR. As an alternative, we used another pair of primers (F11D) that does not cover all the genomes (approximately 10.336 bp).

PCR products were quantified using the fluorimetric method (Qubit^®^ 4 Fluorometer™; Thermo Fisher Scientific, Waltham, MA, United States), and the DNA concentration from each amplicon was adjusted before amplicon pooling (each sample has one pool with all 11 fragments). The DNA concentration of the amplicon pool was adjusted to 0.8 ng/μl to perform the Nextera^®^ XT DNA Sample Library Preparation protocol (Illumina, Inc., San Diego, CA, United States).

The library was purified using AMPure XP^®^ beads (Beckman Coulter™; Life Sciences Division Headquarters; Indianapolis, IN, United States) once quantified and diluted to 2 nM, and denatured according to the manufacturer’s protocol (Preparing DNA Libraries for Sequencing, Miseq Guide). Denatured libraries were loaded in MiSeq Reagent Cartridge v2 (300-cycle) and paired-end sequenced on the MiSeq platform (Illumina, Inc., San Diego, CA, United States).

Nearly complete virus genomes from 40 samples were sequenced with a mean average coverage depth of 5.149× and a breadth coverage ranging from 92.75 to 100% (Supplementary Table 2). Only one sample did not reach quality metrics, and therefore. it was excluded. Phylogenetic analysis was performed using the 39 samples with good quality metrics (average coverage, Q30, and number of reads).

### Sequence Analysis of Yellow Fever Virus Genome

All sequences were trimmed and filtered. Short unpaired reads and low-quality bases and reads were removed using Cutadapt 2.10 (Martin, 2011). Human genome reference was downloaded (GCF_000001405.12), and all unmapped reads were filtered using Samtools (Li et al., 2009), then all FASTQ data were extracted.

FASTQ data were analyzed using SPAdes (Nurk et al., 2013) (trusted contigs using MF538786.2/RJ104 as the sequence reference and *de novo* assembly) and IVA software (Hunt et al., 2015) combining the results to create the most reliable consensus sequence for all samples (Pipeline available at: https://github.com/deyvidamgarten/YFV/wiki/Montagem_genoma_YFV).

The sequence files were downloaded from BaseSpace and checked for quality score (Q30) and trimmed using Cutadapt (Martin, 2011). The next step was mapping and indexing the sequences using BWA (Li and Durbin, 2009) to align our YFV sequences back to the reference. Samtools view (Li et al., 2009) was used to remove the reads with secondary alignment or with low quality of mapping (<30) and/or no mapping at all when compared with the reference in the BAM archive that we had previously generated. Then the sequences were sorted and indexed generating the clean and sorted BAM archive that can be visualized using Integrative Genomics Viewer (IGV) (Thorvaldsdóttir et al., 2013).

Sequence variations in the library were detected using single-nucleotide polymorphism (SNP) and short indel detection function using Freebayes (Garrison and Marth, 2012) and GATK haplotype caller (McKenna et al., 2010) software for each sample, generating a VCF file that then was merged for analysis using BCF tools (Li, 2011).

### Phylogenetic Analysis of Yellow Fever Virus Genomes

Phylogenetic analysis was performed using all the YFV sequences available as complete genomes in NCBI plus those described in Nunes et al. (2012), Gómez et al. (2018), Abreu et al. (2019), Cunha et al. (2019a,b), Delatorre et al. (2019), and Hill et al. (2020). All those complete genomes were included to reconstruct a first phylogeny (complete list of NCBI IDs of YFV genomes used herein are available in the Supplementary Material). A total of 314 genomes plus 39 genomes generated in this study were first analyzed. Sequences with less than 7,000 bp and with more than 1% Ns were removed. The 342 genomes that remained were aligned using MAFFT (Katoh and Standley, 2013), and the region corresponding to positions 143 to 10,309 with respect to NC_002031.1 was used for maximum-likelihood analysis. IQ-TREE2 (Minh et al., 2020) was used for phylogenetic inference. ModelFinder (Kalyaanamoorthy et al., 2017) was used to select the substitution model GTR+F+I+G4 according to BIC. A total of 1,000 replicates of UF-Boot (Hoang et al., 2018) and SH-aLRT (Guindon et al., 2010) was also used to measure consistency and support of nodes. The consensus tree generated in the previous inference (Supplementary Figure 1) was used to determine the available sequences most related to the sequences generated in this study. Thus, the two largest clades near the clade containing the sequences generated in this study (clades A, B, and C in Supplementary Figure 1) were selected for further analysis.

From the 237 sequences belonging to the previously mentioned clades, we removed seven sequences from the Netherlands that were isolated from travelers from Brazil (MK760660, MK760661, MK760662, MK760663, MK760664, MK760665, and MK760666) and other two sequences from Brazil without a collection place information (MF465805 and MH560359). The region corresponding to positions 143 to 10,309 with respect to NC_002031.1 of the 228 remaining sequences were aligned using MAFFT (Katoh and Standley, 2013). The alignment was used for a new maximum-likelihood inference. Again, IQ-TREE2 (Minh et al., 2020) was used for phylogenetic inference. ModelFinder (Kalyaanamoorthy et al., 2017) was used to select the substitution model TIM2+F+I+G4 according to BIC. A total of 1,000 replicates of UF-Boot (Hoang et al., 2018) and SH-aLRT (Guindon et al., 2010) was also used to measure consistency and support of nodes. The NCBI codes and all the available metadata used in this analysis are available in Supplementary Table 3.

### Phylodynamic Analysis of Genomes

The rate of nucleotide substitution, the time to the most recent common ancestors, and the ancestral state reconstruction were estimated using the Markov chain Monte Carlo (MCMC) algorithms implemented in BEAST2 (Bouckaert et al., 2019) with BEAGLE library (Suchard and Rambaut, 2009) to speed up the run time. The same alignment of the 228 sequences previously described was used for this analysis. The evolutionary process was estimated from the sampling year of the sequences (considering the mid of the year of collection) using the GTR substitution model, a strict molecular clock model (Ferreira and Suchard, 2008) or an uncorrelated lognormal molecular clock model (Drummond et al., 2006), and a Bayesian Skyline coalescent tree prior (Drummond et al., 2005). Comparisons among the two clock models were performed using nested sampling with four independent runs for each model (Russel et al., 2019). Migration events throughout the phylogeny were reconstructed using a reversible discrete phylogeographic model (Lemey et al., 2009). A discrete state was assigned for each sequence corresponding to the state (Brazilian sequences) of infection (sequences from our study) or the state reported by the authors (sequences not generated in this study). For the sequences generated in this study, the city/state of YFV infection was collected and mapped using the geolocation tool available at Google Maps^®^ (Supplementary Figure 2) (Google, 2021). MCMC was run sufficiently long to ensure stationarity and convergence. Uncertainty of parameter estimates were assessed after excluding the initial 10% of the run by calculating the effective sample size (ESS) and the 95% highest probability density (HPD) values using TRACER (Rambaut et al., 2018). Tree annotator (Drummond et al., 2012) was used to summarize the posterior tree distribution, and the R package GGTREE (Yu, 2020) was used to visualize and generate the final tree figures.

### Analysis of Synonymous and Non-synonymous Substitutions

For the analysis of synonymous and non-synonymous substitutions, consensus nucleotide sequences were aligned using CLUSTAL W (Larkin et al., 2007). To analyze the presence of these substitutions, the alignment was translated using MEGA7 program (Kumar et al., 2016), Freebayes, and Haplotype Caller software (McKenna et al., 2010; Garrison and Marth, 2012).

## Results

### No Demographic Differences Were Found Between Patients of Different Years

Demographic data analysis from 56 patients enrolled in this study indicates that 85.7% are male aged between 19 and 88 years, old and all of them were RT-qPCR positive for YFV besides its viral load quantified using a standard curve (Casadio et al., 2019). In addition, no significant statistical differences were found after patients were divided into groups considering their respective year of infection (Table 1).

**TABLE 1:** Demographic data of enrolled samples.

Variable	2018 (*n* = 24)	2019 (*n* = 32)	*p*-Value
**Sex**			
Male, *n* (%)	19 (79.2)	29 (90.6)	0.268*^F^*
Female, *n* (%)	5 (20.8)	3 (9.4)	
**Age (years)**			
Mean (min–max)	43.7 (19–74)	45.7 (19–88)	0.596*^U^*
Days after onset***			
Mean (±sd)	5.6 (±2.2)	5.2 (±2.2)	0.536*^U^*
**Viral load (log10)**			
Mean (±sd)	6.3 (±1.32)	6.4 (±1.4)	0.308*^U^*
**Ct**			
Mean (±sd)	27.7 (±4.9)	27.4 (±3.9)	0.328*^U^*

N, number of samples; min, minimum; max, maximum; sd, standard deviation; Ct, cycle threshold; ^F^Fisher exact test, ^U^Mann–Whitney test, p < 0.005; ***before first day of onset.

### Phylogenetic Analysis of Yellow Fever Virus Complete Genomes

The maximum-likelihood tree obtained from 228 YFV complete genomes showed representatives belonging to five genotypes: South American II, South American IB, IC, ID, and IE (Supplementary Figure 3). The monophyletic clade that contains the representatives of ID and IE is well supported (SH-aLRT = 98) (Supplementary Figure 3). A closer look on this monophyletic clade showed well-supported ID and IE sister clades (SH-aLRT = 100 for both ID and IE clades) (Supplementary Figure 4), with all the sequences obtained in this study grouped inside genotype IE (Supplementary Figure 4).

Going deeper in our analysis, we further explore the monophyletic clade IE. This clade can be subdivided into three groups: (i) basal IE, a paraphyletic group with the oldest IE sequences (one sequence from 2002 and one from 2008), (ii) IE1, and (iii) IE2 (all the sequences obtained in the present study belongs to the subclade IE2) (Supplementary Figure 5).

**FIGURE 1:**
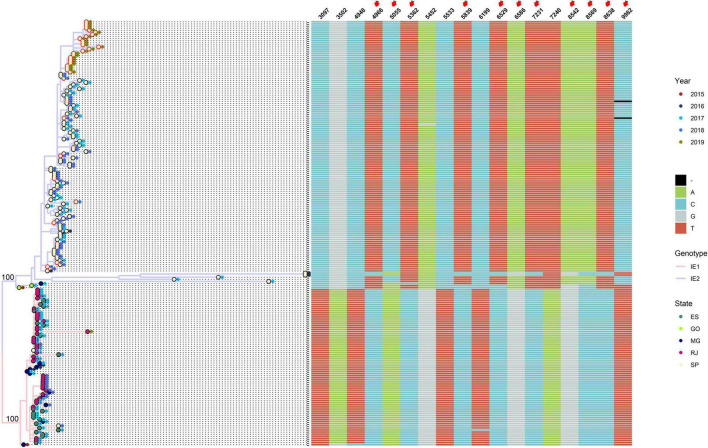
Genotype IE can be subdivided into two major subclades: IE1 and IE2. Maximum-likelihood tree of sequences belonging to genotype IE, showing subdivision in two well-supported clades (SH-aLRT = 100, label of parent nodes), IE1 (pink branches), and IE2 (blue branches). External nodes are colored according to state of collection (internal circle) and year of collection (external circle). Red border in the first circle indicates that the sequence was generated in this study. At the right of the phylogenetic tree, an alignment of relevant positions to discriminate between genotypes is shown.

To determine differences between major clades IE1 and IE2, we integrated to the phylogenetic tree the year and state of collection, and an alignment with the genomic positions that allow us to distinguish between clade IE1 and IE2 (Figure 1). Most of the IE1 sequences were isolated from Espirito Santo (ES), Minas Gerais (MG), or Rio de Janeiro (RJ), just one sequence from this clade was isolated from São Paulo (SP) (Figure 1). On the other hand, almost all IE2 sequences come from SP, and only three sequences were isolated from other states [two from Goias (GO) and one from MG] (Figure 1) (the two sequences from GO in the IE2 clade have an uncertain position, see below).

Although the geographical structure of IE1 and IE2 major clades can be well differentiated, we cannot observe a clear temporal distribution (Figure 1). Both clades contain sequences spanning from 2015 to 2019. Most of the sequences isolated in 2019 belong to the subclade IE2; however, just one 2019 sequence not from this study is available. Because all the sequences from this study were isolated in SP, we cannot see the distribution of genotypes in other states in 2019.

Three genomic positions unambiguously allow us to distinguish between subclades IE1 and IE2 (3,097, 5,533, and 7,240 with respect to the sequence NC_002031.1). The other four positions (3,502, 4,948, 5,452, and 6,199) are distinguishable except for one sequence from one of the subclades (Figure 1). Additionally, the other 11 positions provide differences between subclades IE1 and IE2 but are intermingled in the basal sequences of clade IE2 (Figure 1).

Further subclassification of clade IE1 into IE1_basal, IE1_1, IE1_trans, and IE1_2 based on specific genomic positions are available in Supplementary Figure 6. Subclassification of subclade IE2 into IE2_Basal, IE2_1, IE2_2, IE2_3, and IE2_4 also based on specific genomic position is available in Supplementary Figure 7.

### Phylodynamic Analysis of Major Clades IE1 and IE2

To gain insights in the phylodynamics of the major clades (IE1 and IE2), we performed Bayesian inferences to estimate the time of MRCA and the most probable state of divergence of clades IE1 and IE2. An alignment of 228 complete genomes (see Materials and Methods section) was used to perform this inference. With a strict clock model, the 95% HPD interval estimated for the substitution rate was 2.63E-4–3.42E-4. On the other hand, an uncorrelated lognormal relaxed clock estimated the 95% HPD interval of the mean between 4.21E-4 and 7.90E-4, and the variance between 1.51E-7 and 1.83E-6. To determine which of these models is better adjusted with the data, we used nested sampling (Russel et al., 2019) to estimate the log Bayes factor. Log Bayes factor was 115.31 in favor of the uncorrelated lognormal relaxed clock.

Based on the inference with the uncorrelated lognormal relaxed clock, the divergence time of South America I and South America II genotypes has high margin of uncertainty (1801–1955) (Supplementary Figure 8) with dates in concordance with other studies (Bryant et al., 2007; Auguste et al., 2010; de Souza et al., 2010). This analysis also allows us to estimate the divergence time of genotypes IC (1940–1971), IB (1957–1979), and ID from IE (1978–1992) (Supplementary Figure 8). It was not possible to determine the Brazilian states where those divergences took place (several states appeared with similar probabilities) (Supplementary Figure 8).

**FIGURE 2:**
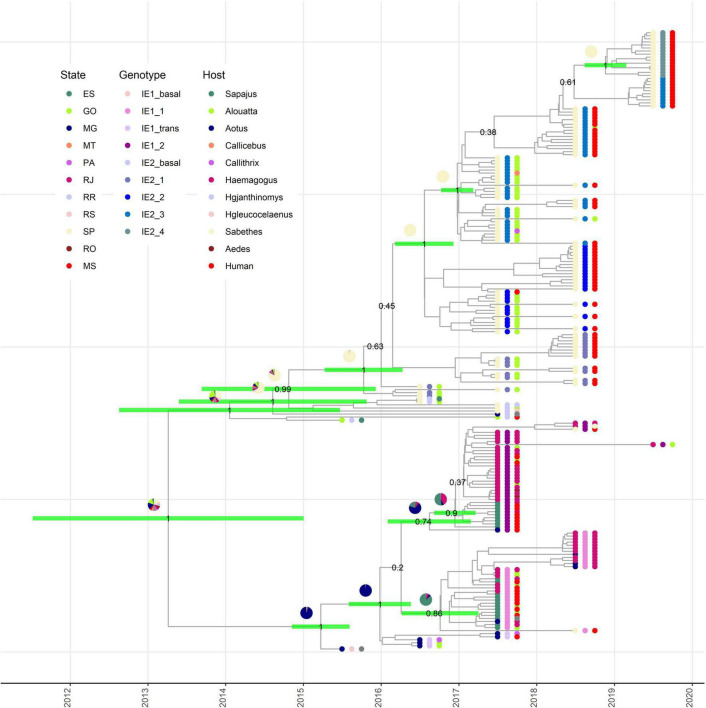
Origin of subclades IE1 and IE2 are revealed by phylodynamic analysis. Time-scaled Bayesian maximum clade credibility tree showing the nodes of divergence between subclades IE1 and IE2 in their respective subdivisions. Green bars in the selected internal nodes show 95% HPD intervals of divergence times. Pie graphics on the internal nodes represent the probability of the state where this node existed. Numbers in the selected internal nodes represent the posterior value. External node points are colored according the state of collection (internal circle), subgenotype (middle circle), and host (external circle).

Our phylodynamic analysis showed that the major clades IE1 and IE2 diverge between mid-2011 and the last months of 2014 (Figure 2). Again, the state where this divergence took place could not be estimated with certainty (Figure 2). Additionally, this analysis permits us to determine that the MRCA for clade IE1 exists between the last months of 2014 and mid-2015 in MG (Figure 2). The MRCA of the subclade IE1_1 was estimated to exist between mid-2016 and the first months of 2017 in ES (Figure 2). In contrast, the subclade IE1_2 have its origin in MG from where it was introduced to ES and RJ between the last months of 2016 and the first months of 2017 (Figure 2).

The MRCA of the major clade IE2 existed between the last months of 2012 and mid-2015, but the state of origin is uncertain, with highest probabilities for SP and GO (Figure 2). After its appearance, it is clear that it continues diverging in subclades in SP (Figure 2). The four IE2 subclades described above also appear well supported in our phylodynamic analysis. Thus, our analysis estimated that all these subclades have their origin in SP. The subclade IE2_1 diverged between the first months of 2015 and the first months of 2016, IE2_2 during 2016, IE2_3 between the last months of 2016 and the first months of 2017, and the most recent subclade, IE2_4, between the last months of 2018 and the beginning of 2019.

These results support the hypothesis that a single lineage introduced to SP gave rise to the establishment of the IE2 clade in SP (Figure 2). On the other hand, just one sequence of IE1 subclade has been described in SP in 2018, and this observation confirms an independent introduction of subclade IE1 to SP, but apparently IE1 clade has not dispersed in SP as effectively as IE2. At the moment, any of the IE2 subclades has been found in a state different from SP.

All the IE1 and IE2 subclades (except IE2_4) described here have at least one representant isolated from NHPs or mosquitoes. Thus, these subclades could arise in humans, NHPs, or mosquitoes. Anyway, this is a clear evidence of frequent interchange between human and NHPs YFVs.

### Synonymous and Non-synonymous Substitutions Analysis

From the substitution analysis, we found 46 nucleotide substitutions, including 20 non-synonymous substitutions in the amino acid level: three in the capsid protein (K26R, I43V, and K60R), four in the envelope (H301Y, A341V, N555D, and D597G), three in the NS1 (Y953H, L994P, and G1067R), one in the NS2A (A1209V), three in the NS3 (N1646T, T1826M, and P1953H), two in the NS4A (V2136G and L2137P), and four in the NS5 (R2535W, M2620V, A3149V, and T3229I). Only N1646T in the NS3 are present in all the 39 samples, as an SNP signature for these outbreaks. Furthermore, the substitution T3329I in the NS5 is present in almost all sequences (30/39), and K26R in capsid protein is present only in 15/29 sequences, all of them from the 2019 strain (Supplementary Table 4).

## Discussion

In this study, we analyzed 56 yellow fever virus patients and generated 39 new YFV nearly complete genomic sequences from samples from humans, collected in HCFMUSP during the 2018 and 2019 outbreaks. First, we conducted a demographic data analysis indicating a homogeneity between the 2018 and 2019 sample groups. In accordance with the disease monitoring carried out by Brazil’s Ministry of Health, we report a higher percentage of YF in men (82.1%) since it is considered a reflection of the work activities performed by them in or near forest areas (Vasconcelos, 2003).

Phylogenetic analysis was made corroborating the fact that all 39 sequences belong to the South American IE. In order to determine the existence of possible subclades, we analyzed a phylogenetic inference of 228 YFV complete genomes. Similar to that describe by Delatorre et al. (2019), we proposed that the IE subclade can be further divided into two major clades: IE1 and IE2 [named YFV_*MG/ES/RJ*_ and YFV_*MG/SP*_, respectively, by Delatorre et al. (2019)]. We renamed those clades as IE1 and IE2 to allow easy naming of new subclades as IE1_1, IE1_2, IE2_1, IE2_2, IE2_3, and IE2_4 described here. Based on this analysis, we classified the genomes generated in this study as belonging to IE2 with representants of all four subclades (Supplementary Table 3).

Phylodynamic analyses showed a strong geographical structure of the major clades IE1 and IE2. However, our analysis was not able to determine the state of divergence of these major clades. Delatorre et al. (2019) mentioned GO as the most likely state (0.57 probability) where this divergence took place. However, no sequence of 2016 from SP was included in that study. The inclusion of several sequences from SP in our study increases the uncertainty of the state of divergence of the major clades IE1 and IE2 (Figure 2). On the other hand, our estimations of the date of divergence of these clades are similar to those described by Delatorre et al. (2019) (2011–2015) and inside the 95% HPD interval (2014–2016) mentioned by Rezende et al. (2018) inferred from 1,038-nt sequences.

Our deeper analysis of clade IE1 showed that it originated in MG from where it was introduced to ES to form the subclade IE1_1. From here, subclade IE1_1 was dispersed to RJ and returned to MG (Figure 2). These results are in accordance with one of the subclades of IE1 (YFV_*MG/ES/RJ*_) as shown by Delatorre et al. (2019). In the case of subclade IE1_2, Delatorre et al. (2019) indicated its origin in ES from where it was introduced to RJ. However, Delatorre et al. (2019) did not include the genome from MG with NCBI code MF370533 that in our analysis appeared basal to subclade IE1_2 (Figure 2). The inclusion of this genome modifies the origin of this subclade and established the origin in MG from where it moves to ES and RJ (Figure 2). Dates of the MRCA of the new proposed subclades IE1_1 and IE1_2 match with the report of Delatorre et al. (2019) (Figure 2).

Cunha et al. (2019a) hypothesized that the major clade IE2 (or YFV_*MG/SP*_) originated in MG. However, their inference was done without any genome of neither 2016–2017 SP nor GO. In contrast, the study of Hill et al. (2020) that included earlier SP genomes and GO genomes were not able to accurately determine the state of the MRCA of IE2. Our analysis, which also includes GO and early SP genomes, cannot accurately determine the state of origin of IE2, but showed GO and SP as the most likely states with similar probabilities (Figure 2). Importantly, we were able to accurately determine the state of origin of the new proposed subclades IE2_1, IE2_2, IE2_3, and IE2_4 and their respective times of divergence (Figure 2).

Subclades IE2_3 and IE2_4 were only observed during 2019. If this is a sample bias or if this is the process of lineage replacement in SP is an open question that has to be answered in the next studies.

We analyzed the 39 YFV genomes generated in this study, searching for synonymous and nonsynonymous mutations among them. Nine of the non-synonymous substitutions involve changes in the amino acid functional classes. Interestingly, two of these nine amino acid changes are located in two important proteins of the viral replicase complex: NS3 protein with RNA helicase, serine protease (Chen et al., 2017), and nucleoside triphosphatase (NTPase) (Brand et al., 2017) domains and NS5 protein, the largest and highly conserved protein in flavivirus considered a key for viral replication (Baleotti et al., 2003). Amino acid changes in these conserved proteins may have an impact on viral infectivity, both in humans and NHPs, as well as in mosquitoes (Gómez et al., 2018).

Comparing the 2018 and 2019 sequences, it was possible to observe a non-synonymous mutation at nucleotide 195 (K26R) in the capsid protein, which occurs in 15/25 samples in 2019, but found in just one YFV genome from 2018 (not from this study). This mutation, together with mutations in positions 2,545, 2,623, and 9,406 are the fingerprint of IE2_4. Studies associate the capsid protein with the packaging of the viral genome and the formation of the nucleocapsid (Patkar et al., 2007).

Aiming to explore more about the genomes that were sequenced in this study, we analyzed if patients who were also attended in HCFMUSP and evolved to death had similar variants when compared with those who survived. For this goal, we explored the studies made by Cunha et al. (2019a) that sequenced 36 YFV whole genomes from patients who evolved to death. Phylogenetic analysis did not find specific clades with higher percentages of patients that evolved to death.

These findings reinforce the idea that continued genomic surveillance strategies are needed to assist in the monitoring and understanding of YFV epidemics aiming to help public health actions and the management of infections. As shown here, inclusion of new genomes can update our hypotheses and confirm those of others helping to better understand the epidemiology of YFV.

Monitoring of the new subclades described here could help in determining interconnections between southern states. It is intriguing why clades IE1 and IE2 have a strong geographical structure despite the high human transit between the southern states, especially RJ and SP. YFV strains from NHPs from Brazil’s southeast are necessary to determine if the subclade IE2_4 here described has been maintained in cycles in humans and/or NHPs.

## Data Availability Statement

The datasets presented in this study can be found in online repositories. The names of the repository/repositories and accession number(s) can be found below: NCBI GenBank MZ604838–MZ604876.

## Ethics Statement

The studies involving human participants were reviewed and approved by this study was conducted in compliance with institutional guidelines, approved by the ethical committee from the Hospital das Clínicas da Faculdade de Medicina da Universidade de São Paulo (CEP/HCFMUSP; CAAE: 74535417.3,1001.0068) and all individuals signed written informed consent forms. The patients/participants provided their written informed consent to participate in this study.

## Author Contributions

AM, FM, and DE conceived and designed the experiments. FM and MS performed the experiments. Y-LH and LV were essential to the data collection. SJ performed the phylogenetic inferences. DE, FM, and SJ analyzed the data. FM and SJ drafted the work. DE, JR, FJ, SJ, and Y-LH revised the manuscript critically for important intellectual content. All authors contributed to the article and approved the submitted version.

## Conflict of Interest

The authors declare that the research was conducted in the absence of any commercial or financial relationships that could be construed as a potential conflict of interest.

## Publisher’s Note

All claims expressed in this article are solely those of the authors and do not necessarily represent those of their affiliated organizations, or those of the publisher, the editors and the reviewers. Any product that may be evaluated in this article, or claim that may be made by its manufacturer, is not guaranteed or endorsed by the publisher.

## Abbreviations

YF: yellow fever
YFV: yellow fever virus
PCR: polymerase chain reaction
NGS: next-generation sequencing
NHPs: non-human primates
UTR: untranslated region
ORF: open reading frame
IGV: Integrative Genomics Viewer
SNP: single nucleotide polymorphism
NTPase: nucleoside triphosphatase.
