# Supplementary material for: Updating the Phylodynamics of Yellow Fever Virus 2016–2019 Brazilian Outbreak With New 2018 and 2019 São Paulo Genomes
Source: Front Microbiol. 2022 Apr 14;13:811318. doi: 10.3389/fmicb.2022.811318 (PMC9132216; doi:10.3389/fmicb.2022.811318)
Supplement: Supplementary file 1 [file Data_Sheet_1.docx]

**Figure S1. Maximum likelihood tree of 353 yellow fever virus complete genomes.** The internal node that contains clade A, B, and C is labelled by its SH-aLRT value. The sequences of the three labelled clades (A, B, and C) were used for the subsequent analysis.Red border in the tip circle indicates that the sequence was generated in this study.

**Figure S2. Mapping of YFV probable infection sites. Analysis was performed using Google Maps**® (44) tools available at the platform. This map highlights the cities identified during data collection from enrolled patients in this study. Data were divided into cities and their respective outbreak year. Only one patient from the 2019 outbreak indicates the infection site as being Mairipora.

**Figure S3. Maximum likelihood tree of 228 yellow fever virus complete genomes.** Tips are colored according to their corresponding genotypes.The internal node containing genotypes IE and ID is labelled by its SH-aLRT value. Red border in the tip circle indicates that the sequence was generated in this study.

**Figure S4. ID and IE subtree of the ML tree of 228 yellow fever virus complete genomes.** A) ML tree showing the distribution of YFV by year of collection (color of tips) or B) state of collection. The internal nodes that represents the MRCA of clade ID and IE are labelled by its SH-aLRT value. Red border in the tip circle indicates that the sequence was generated in this study.

**Figure S5. IE subtree of the ML tree of 228 yellow fever virus complete genomes.** A) ML tree showing the distribution of YFV belonging to genotype IE by year of collection (color of tips) or B) state of collection. The internal nodes that before the divergence of subgenotypes IE1 and IE2 are colored according its SH-aLRT value. Red border in the tipcircle indicates that the sequence was generated in this study.

**Figure S6. Subdivision of genotype IE1.**Maximum likelihood subtree of sequences belonging to subgenotype IE1. Well-supported nodes (SH-aLRT> 85, label of nodes) divide IE1 into two major subgenotypes (IE1_1 and IE1_2) and two other groups that possibly represent transitions between major subgenotypes (IE1_basal and IE1_trans). Branches are colored according to subgenotype. External nodes are colored according to state of collection (internal circle) and year of collection (external circle). At the right of the phylogenetic tree, an alignment of relevant positions to discriminate between subgenotypes is showed.

**Figure S7. Subdivision of genotype IE2.**Maximum likelihood subtree of sequences belonging to subgenotype IE2. Well-supported nodes (SH-aLRT>95, label of nodes) divide IE2 into four major subgenotypes (IE2_1 to IE2_4) and a basal group (IE2_basal). Branches are colored according to subgenotype. External nodes are colored according to state of collection (internal circle) and year of collection (external circle). Red border in the first circle indicates that the sequence was generated in this study.At the right of the phylogenetic tree, an alignment of relevant positions to discriminate between subgenotypes is showed.

**Figure S8. Phylodynamic of YFV complete genomes.**Time-scaled Bayesian maximum clade credibility tree showing the nodes of divergence between genotypes included in this study. Green bars in the selected internal nodes shows 95 HPD intervals of divergence times. Pie graphics on the internal nodes represents the probability of the state where this node existed. Numbers in the selected internal nodes represent the posterior value. External nodes are colored according the subgenotype (internal circle). Subgenotypes IE1 and IE2 (without considering IE1_basal and IE_basal) are collapsed to improve visualization.
