# Supplementary material for: Updating the Phylodynamics of Yellow Fever Virus 2016–2019 Brazilian Outbreak With New 2018 and 2019 São Paulo Genomes
Source: Front Microbiol. 2022 Apr 14;13:811318. doi: 10.3389/fmicb.2022.811318 (PMC9132216; doi:10.3389/fmicb.2022.811318)
Supplement: Supplementary file 2 [file Data_Sheet_2.docx]

Supplementary Material

**Supplementary Table I**

**Primers sequences (n=12) for the YFV sequencing scheme.** nt = nucleotide; F and L: primer forward; R: primer reverse

| **Fragments** | **nt positon** | **Primer** | **Sequence 5'- 3'** | **Amplicon size** | **Reference** |
| --- | --- | --- | --- | --- | --- |
| F1 | 28 | 28F | CATTGGTCTGCAAATCGAGTTG | 1,120pb | Present study |
|  | 1148 | 1148R | TTCATTGAGGGGGTGCATGG |  |  |
| F2 | 1004 | 1004F | TYAAGACTGCACTGTARCACAC | 1,131pb | Present study |
|  | 2135 | 2135R | TGATGCARGTGAARGTGCCA |  |  |
| F3 | 1929 | 1929F | TRCCYTCCTTGTGCCACTG | 1,136pb | Present study |
|  | 3065 | 3065R | CYCGAGTGTAYATGGATGCAG |  |  |
| F4 | 2946 | 2946F | ATCCATGTTCCATTCACCTCATG | 1,230pb | Present study |
|  | 4176 | 4176R | AATGGYTGAGGTGAGACTTGC |  |  |
| F5 | 3981 | 11L | TGACAATGGCTGAGGTGAGACT | 1,208pb | Faria N.R et al.,2018 |
|  | 5189 | 13R | GTCTTGTTTTCCCAGCTCCAGG |  |  |
| F6 | 5080 | 14L | AACTGAGGTGAAAGAGGAGGGG | 1,197pb | Faria N.R et al.,2018 |
|  | 6277 | 16R | CTCAAAGCACCACTTTCGGTCA |  |  |
| F7 | 6191 | 6191F | CTCTCCACCYTTCTTGGCCA | 1,150pb | Present study |
|  | 7341 | 7341R | GTGGCTGGAATTCAATAACRGTG |  |  |
| F8 | 7182 | 7182F | TTCCTCRATGTCRACTGTTGGA | 1,175pb | Present study |
|  | 8357 | 8357R | GTGTGAAGGTGTTGGCTCCA |  |  |
| F9 | 8175 | 8175F | GGRCGCCTCATTCTYCTCAT | 1,289pb | Present study |
|  | 9464 | 9464R | ATGACACAGCTGGATGGGAC |  |  |
| F10 | 9237 | 9237F | GTTGGTGATGGTRTTCAARGC | 875pb | Present study |
|  | 10112 | 10112R | TGGCTGTTTCTTCTGCTGTTC |  |  |
| F11 | 9978 | 9978F | GTTTTGUCCTGCATGTGTGG | 989pb | Present study |
|  | 10967 | 10967R | TCTTTCCCTGGCGTCAATATG |  |  |
| F11 D | 9114 | 25L | TGAATGAGGACCACTGGGCATC | 1,222pb | Faria N.R et al.,2018 |
|  | 10336 | 27R | CTGCAGATCAGCATCCACAGAG |  |  |

**Supplementary Table II**

**Information about the samples enrolled in this study and statistics for the sequences generated using Illumina MiSeq sequencer.** *death after dischargedbp: base pairs; (-) without information; RT-PCR: Reverse Transcriptase Real timePCR; Ct: threshold cycle.

| **Patient ID** | **Outcome** | **Days after Onset** | **RT-qPCR (Ct)** | **Material** | **Genome Coverage bp (%)** | **Average Coverage** | **Genbank** |
| --- | --- | --- | --- | --- | --- | --- | --- |
| SP01/BRA/2018 | Discharged | 5 | 20.6 | serum | 10279 (93.4) | 5,843 | MZ604840 |
| SP02/BRA/2018 | Discharged | 7 | 31.7 | serum | 10266 (93.3) | 5,279 | MZ604868 |
| SP03/BRA/2018 | Discharged | 4 | 29.9 | serum | 10269 (93.3) | 5,574 | MZ604842 |
| SP04/BRA/2018 | Discharged | 10 | 31.3 | serum | 10322 (93.8) | 4,305 | MZ604845 |
| SP05/BRA/2018 | Discharged | 6 | 28 | serum | 10268 (93.3) | 7,221 | MZ604870 |
| SP06/BRA/2018 | Discharged | 3 | 16.3 | serum | 10849 (98.5) | 5,899 | MZ604856 |
| SP07/BRA/2018 | Discharged | 3 | 23.9 | serum | 10279 (93.4) | 4,974 | MZ604859 |
| SP08/BRA/2018 | Discharged | 5 | 30.3 | serum | 10275 (93.4) | 5,522 | MZ604861 |
| SP09/BRA/2018 | Discharged | 6 | 33.1 | serum | 10852 (98.6) | 4,553 | MZ604872 |
| SP10/BRA/2018 | Discharged | 2 | 15 | serum | 10279 (93.4) | 4,791 | MZ604862 |
| SP11/BRA/2018 | Discharged | 4 | 29.9 | serum | 10187 (92.5) | 4,144 | MZ604873 |
| SP12/BRA/2018 | Discharged | 4 | 24.2 | serum | 10835 (98.4) | 6,397 | MZ604865 |
| SP13/BRA/2018 | Discharged | 9 | 29 | urine | 10839 (98.5) | 3,66 | MZ604866 |
| SP14/BRA/2018 | Discharged | 4 | 28 | serum | 10269 (93.3) | 4,414 | MZ604876 |
| SP01/BRA/2019 | Discharged | 3 | 28.4 | urine | 10266 (93.3) | 5,088 | MZ604838 |
| SP02/BRA/2019 | Discharged | 3 | 24.2 | serum | 10279 (93.4) | 4,418 | MZ604839 |
| SP03/BRA/2019 | Discharged | 10 | 20 | urine | 10279 (93.4) | 5,573 | MZ604841 |
| SP04/BRA/2019 | Discharged | 7 | 30.2 | serum | 10269 (93.3) | 4,91 | MZ604869 |
| SP05/BRA/2019 | Discharged | 10 | 25.6 | urine | 10080 (91.6) | 5,472 | MZ604843 |
| SP06/BRA/2019 | Discharged | 4 | 22.5 | urine | 10269 (93.3) | 6,998 | MZ604844 |
| SP07/BRA/2019 | Discharged | 2 | 19.8 | serum | 10859 (98.6) | 6,557 | MZ604846 |
| SP08/BRA/2019 | Discharged | 4 | 31.6 | urine | 10269 (93.3) | 3,729 | MZ604847 |
| SP09/BRA/2019 | Discharged | 7 | 34.1 | urine | 10862 (98.7) | 7,348 | MZ604848 |

**Supplementary Table II (continued)**

**Information about the samples enrolled in this study and statistics for the sequences generated using Illumina MiSeq sequencer.** *death after discharged bp: base pairs; (-) without information; RT-PCR: Reverse Transcriptase Real timePCR; Ct: threshold cycle.

| **Patient ID** | **Outcome** | **Days after Onset** | **RT-qPCR (Ct)** | **Material** | **Genome Coverage bp (%)** | **Average Coverage** | **Genbank** |
| --- | --- | --- | --- | --- | --- | --- | --- |
| SP10/BRA/2019 | Discharged | 7 | 28.2 | urine | 10860 (98.7) | 7,752 | MZ604849 |
| SP11/BRA/2019 | Discharged | 5 | 33.9 | urine | 10279 (93.4) | 6,153 | MZ604850 |
| SP12/BRA/2019 | Discharged | 7 | 26.8 | urine | 10277 (93.4) | 4,127 | MZ604851 |
| SP13/BRA/2019 | Discharged | 3 | 27.9 | serum | 10269 (93.3) | 3,675 | MZ604852 |
| SP14/BRA/2019 | Discharged | 6 | 19.6 | urine | 10855 (98.6) | 2,861 | MZ604853 |
| SP15/BRA/2019 | Discharged | 3 | 27.9 | urine | 10188 (92.5) | 5,728 | MZ604855 |
| SP16/BRA/2019 | Discharged | 3 | 27.1 | serum | 10855 (98.6) | 5,141 | MZ604858 |
| SP17/BRA/2019 | Discharged | 6 | 26.5 | urine | 10252 (93.1) | 1,675 | MZ604860 |
| SP18/BRA/2019 | Discharged | 6 | 29.1 | urine | 10257 (93.2) | 3,426 | MZ604863 |
| SP19/BRA/2019 | Discharged | 5 | 23.9 | urine | 10853 (98.6) | 2,678 | MZ604864 |
| SP20/BRA/2019 | Discharged | 4 | 29.2 | urine | 10283 (93.4) | 6,073 | MZ604874 |
| SP21/BRA/2019 | Discharged | 6 | 29 | urine | 10271 (93.3) | 4,840 | MZ604875 |
| SP22/BRA/2019 | Discharged | 4 | 30.8 | urine | 10853 (98.6) | 1,871 | MZ604867 |
| SP23/BRA/2019 | Discharged* | 4 | 20.4 | urine | 10855 (98.6) | 6,267 | MZ604854 |
| SP24/BRA/2019 | Discharged* | 6 | 25.5 | urine | 10644 (96.7) | 4,108 | MZ604871 |
| SP25/BRA/2019 | Discharged* | 4 | 26.5 | urine | 10849 (98.6) | 7,769 | MZ604857 |
| SP26/BRA/2019 | Discharged | 5 | 30.2 | urine | - | - |  |

**Supplementary Table III: Complete YFV genomes sequences used in the phylogenetic analysis.**(-)withoutinformation

| **NCBI accession number** | **Local** | **Year** | **Brazilian State** | **Host** | **Country** | **Subgenotype** | **Genotype** | **Subclade** | **Classification Suggestion** |
| --- | --- | --- | --- | --- | --- | --- | --- | --- | --- |
| HM582851 | - | 2009 | - | Alouatta | Trinidad_and_Tobago | - | South America I | - | - |
| MF004382 | - | 1999 | - | Human | Bolivia | II | South America II | II | II |
| KY861728 | Caibate | 2008 | RS | Alouatta | Brazil | IE | South America I | IE | IE |
| MF370530 | Alvarenga | 2017 | MG | Haemagogus | Brazil | IE | South America I | IE1 | IE1_1 |
| MF370531 | Vitoria | 2017 | ES | Human | Brazil | IE | South America I | IE1 | IE1_1 |
| MF370532 | Sao Francisco | 2017 | MG | Human | Brazil | IE | South America I | IE1 | IE1_trans |
| MF370533 | Ladainha | 2017 | MG | Human | Brazil | IE | South America I | IE1 | IE1_2 |
| MF370534 | Mirabela | 2016 | MG | Callithrix | Brazil | IE | South America I | IE1 | IE1_trans |
| MF370535 | Januarua | 2016 | MG | Alouatta | Brazil | IE | South America I | IE1 | IE1_trans |
| MF370536 | Conego | 2016 | MG | Alouatta | Brazil | IE | South America I | IE1 | IE1_trans |
| MF370537 | Laranja da Terra | 2017 | ES | - | Brazil | IE | South America I | IE1 | IE1_1 |
| MF370538 | Vigem da Lapa | 2017 | MG | Callithrix | Brazil | IE | South America I | IE1 | IE1_trans |
| MF370539 | Alenquer | 2017 | PA | Sapajus | Brazil | IE | South America I | IE | IE |
| MF370540 | Rorainopolis | 2017 | RR | Alouatta | Brazil | IE | South America I | IE | IE |
| MF370541 | Oriximina | 2017 | PA | Alouatta | Brazil | IE | South America I | IE | IE |
| MF370542 | Monte Alegra | 2017 | PA | Alouatta | Brazil | IE | South America I | IE | IE |
| MF370543 | Sao Jose do Povo | 2017 | MT | Alouatta | Brazil | IE | South America I | IE | IE |
| MF370544 | Santarem | 2017 | PA | Aotus | Brazil | IE | South America I | IE | IE |
| MF370545 | Tucurui | 2017 | PA | Aotus | Brazil | ID | South America I | ID | ID |
| MF370546 | Novo Repartimento | 2017 | PA | Alouatta | Brazil | IE | South America I | IE | IE |
| MF370547 | Sao Sebastiao do Alto | 2017 | RJ | Alouatta | Brazil | IE | South America I | IE1 | IE1_2 |
| MF370548 | Carmo | 2017 | RJ | Alouatta | Brazil | IE | South America I | IE1 | IE1_1 |
| MF370549 | Natalandia | 2015 | RJ | - | Brazil | IE | South America I | IE1 | IE1_basal |
| MF170968 | Marechal Floriano | 2017 | ES | Human | Brazil | IE | South America I | IE1 | IE1_1 |
| MF170969 | Casimiro de Abreu | 2017 | ES | Human | Brazil | IE | South America I | IE1 | IE1_2 |
| MF170970 | Domingo Martins | 2017 | ES | Human | Brazil | IE | South America I | IE1 | IE1_2 |
| MF170971 | Sao Roque de Minas | 2017 | MG | - | Brazil | IE | South America I | IE2 | IE2_basal |
| MF170972 | Cariacica | 2017 | ES | Human | Brazil | IE | South America I | IE1 | IE1_2 |

**Supplementary Table III (continued)**

| MF170973 | Domingo Martins | 2017 | ES | Human | Brazil | IE | South America I | IE1 | IE1_2 |
| --- | --- | --- | --- | --- | --- | --- | --- | --- | --- |
| MF170974 | Cariacica | 2017 | ES | Human | Brazil | IE | South America I | IE1 | IE1_2 |
| MF170975 | Brejetuba | 2017 | ES | Human | Brazil | IE | South America I | IE1 | IE1_1 |
| MF170976 | Laranja da Terra | 2017 | ES | Human | Brazil | IE | South America I | IE1 | IE1_1 |
| MF170977 | Conceicao do Castelo | 2017 | ES | Human | Brazil | IE | South America I | IE1 | IE1_1 |
| MF170978 | Santa Maria de Jetiba | 2017 | ES | Human | Brazil | IE | South America I | IE1 | IE1_1 |
| MF170979 | Santa Maria de Jetiba | 2017 | ES | Human | Brazil | IE | South America I | IE1 | IE1_1 |
| MF170980 | Alfredo Chaves | 2017 | ES | Human | Brazil | IE | South America I | IE1 | IE1_2 |
| MF170981 | Santa Leopoldina | 2017 | ES | Human | Brazil | IE | South America I | IE1 | IE1_2 |
| MG969501 | Bom Despacho | 2001 | MG | Human | Brazil | ID | South America I | ID | ID |
| MK533792 | Casimiro de Abreu | 2019 | RJ | Alouatta | Brazil | IE | South America I | IE1 | IE1_2 |
| MW034590 | Jaboticabal | 2016 | SP | Alouatta | Brazil | IE | South America I | IE2 | IE2_1 |
| MK583147 | Mairipora | 2018 | SP | Human | Brazil | IE | South America I | IE2 | IE2_3 |
| MK583148 | Mairipora | 2018 | SP | Human | Brazil | IE | South America I | IE2 | IE2_3 |
| MK583149 | Sao Paulo | 2018 | SP | Human | Brazil | IE | South America I | IE2 | IE2_3 |
| MK583150 | Itaquaquecetuba | 2018 | SP | Human | Brazil | IE | South America I | IE2 | IE2_1 |
| MK583151 | Mairipora | 2018 | SP | Human | Brazil | IE | South America I | IE2 | IE2_3 |
| MK583152 | Ibiuna | 2017 | SP | Human | Brazil | IE | South America I | IE2 | IE2_2 |
| MK583153 | Mairipora | 2018 | SP | Human | Brazil | IE | South America I | IE2 | IE2_3 |
| MK583154 | Sao Paulo | 2018 | SP | Human | Brazil | IE | South America I | IE1 | IE1_1 |
| MK583155 | Mairipora | 2018 | SP | Human | Brazil | IE | South America I | IE2 | IE2_3 |
| MK583156 | Atibaia | 2018 | SP | Human | Brazil | IE | South America I | IE2 | IE2_2 |
| MK583157 | Guarulhos | 2018 | SP | Human | Brazil | IE | South America I | IE2 | IE2_3 |
| MK583158 | Mairipora | 2018 | SP | Human | Brazil | IE | South America I | IE2 | IE2_3 |
| MK583159 | Atibaia | 2018 | SP | Human | Brazil | IE | South America I | IE2 | IE2_1 |
| MK583160 | Cotia | 2018 | SP | Human | Brazil | IE | South America I | IE2 | IE2_2 |
| MK583161 | Itaquaquecetuba | 2018 | SP | Human | Brazil | IE | South America I | IE2 | IE2_2 |

**Supplementary Table III (continued)**

| MK583162 | Sao Paulo | 2018 | SP | Human | Brazil | IE | South America I | IE2 | IE2_2 |
| --- | --- | --- | --- | --- | --- | --- | --- | --- | --- |
| MK583163 | Sao Paulo | 2018 | SP | Human | Brazil | IE | South America I | IE2 | IE2_3 |
| MK583164 | Cotia | 2018 | SP | Human | Brazil | IE | South America I | IE2 | IE2_2 |
| MK583165 | Mairipora | 2018 | SP | Human | Brazil | IE | South America I | IE2 | IE2_3 |
| MK583166 | Sao Paulo | 2018 | SP | Human | Brazil | IE | South America I | IE2 | IE2_3 |
| MK583167 | Guarulhos | 2018 | SP | Human | Brazil | IE | South America I | IE2 | IE2_3 |
| MK583168 | Sao Paulo | 2018 | SP | Human | Brazil | IE | South America I | IE1 | IE1_2 |
| MK583169 | Guarulhos | 2018 | SP | Human | Brazil | IE | South America I | IE2 | IE2_1 |
| MK583170 | Guarulhos | 2018 | SP | Human | Brazil | IE | South America I | IE2 | IE2_1 |
| MK583171 | Guarulhos | 2018 | SP | Human | Brazil | IE | South America I | IE2 | IE2_1 |
| MK583172 | Guarulhos | 2018 | SP | Human | Brazil | IE | South America I | IE2 | IE2_2 |
| MK583173 | Guarulhos | 2018 | SP | Human | Brazil | IE | South America I | IE2 | IE2_1 |
| MK583174 | Aruja | 2018 | SP | Human | Brazil | IE | South America I | IE2 | IE2_1 |
| MK583175 | Itaquaquecetuba | 2018 | SP | Human | Brazil | IE | South America I | IE2 | IE2_2 |
| MK583176 | Piedade | 2018 | SP | Human | Brazil | IE | South America I | IE2 | IE2_2 |
| MK583177 | Guarulhos | 2018 | SP | Human | Brazil | IE | South America I | IE2 | IE2_2 |
| MK583178 | Itariri | 2018 | SP | Human | Brazil | IE | South America I | IE2 | IE2_2 |
| MK583179 | Ibiuna | 2018 | SP | Human | Brazil | IE | South America I | IE2 | IE2_2 |
| MK583180 | Guarulhos | 2018 | SP | Human | Brazil | IE | South America I | IE2 | IE2_1 |
| MK583181 | Sao Lourenco da Serra | 2018 | SP | Human | Brazil | IE | South America I | IE2 | IE2_2 |
| MK583182 | Guarulhos | 2018 | SP | Human | Brazil | IE | South America I | IE2 | IE2_2 |
| MH666056 | Catanduva | 2016 | SP | Alouatta | Brazil | IE | South America I | IE2 | IE2_basal |
| MH666057 | Ribeirao Preto | 2017 | SP | Alouatta | Brazil | IE | South America I | IE2 | IE2_basal |
| MH666058 | Tabapua | 2016 | SP | Sapajus | Brazil | IE | South America I | IE2 | IE2_basal |
| MH666059 | Ribeirao Preto | 2017 | SP | Hgleucocelaenus | Brazil | IE | South America I | IE2 | IE2_basal |
| MH666060 | Ribeirao Preto | 2017 | SP | Hgjanthinomys | Brazil | IE | South America I | IE2 | IE2_basal |
| MK333804 | Novo Brasil | 2015 | GO | Sapajus | Brazil | IE | South America I | IE2 | IE2_basal |
| MK333803 | Nova Crixas | 2017 | GO | Alouatta | Brazil | IE | South America I | IE | IE |

**Supplementary Table III (continued)**

| MK728873 | Amorinopolis | 2017 | GO | Human | Brazil | IE | South America I | IE2 | IE2_basal |
| --- | --- | --- | --- | --- | --- | --- | --- | --- | --- |
| MK333807 | Belmiro Braga | 2018 | MG | Haemagogus | Brazil | IE | South America I | IE1 | IE1_1 |
| MK333808 | Belmiro Braga | 2018 | MG | Haemagogus | Brazil | IE | South America I | IE1 | IE1_1 |
| MK333806 | JuizdeFora | 2018 | MG | Haemagogus | Brazil | IE | South America I | IE1 | IE1_1 |
| MK333800 | Domingo Martins | 2017 | ES | Alouatta | Brazil | IE | South America I | IE1 | IE1_1 |
| MK333802 | Santa Leopoldina | 2017 | ES | Haemagogus | Brazil | IE | South America I | IE1 | IE1_2 |
| MK333801 | Santa Leopoldina | 2017 | ES | Haemagogus | Brazil | IE | South America I | IE1 | IE1_2 |
| MK333809 | Valenca | 2018 | RJ | Haemagogus | Brazil | IE | South America I | IE1 | IE1_1 |
| MK333805 | IlhaGrande | 2018 | RJ | Sabethes | Brazil | IE | South America I | IE1 | IE1_2 |
| MK333798 | Sao Paulo | 2018 | SP | Human | Brazil | IE | South America I | IE2 | IE2_3 |
| MK333799 | Sao Paulo | 2018 | SP | Human | Brazil | IE | South America I | IE2 | IE2_3 |
| MF423373 | Domingo Martins | 2017 | ES | Haemagogus | Brazil | IE | South America I | IE1 | IE1_1 |
| MF423374 | Domingo Martins | 2017 | ES | Haemagogus | Brazil | IE | South America I | IE1 | IE1_1 |
| KY885000 | Domingo Martins | 2017 | ES | Alouatta | Brazil | IE | South America I | IE1 | IE1_1 |
| KY885001 | Domingo Martins | 2017 | ES | Alouatta | Brazil | IE | South America I | IE1 | IE1_1 |
| MF423375 | Macae | 2017 | RJ | Alouatta | Brazil | IE | South America I | IE1 | IE1_2 |
| MF423376 | Macae | 2017 | RJ | Alouatta | Brazil | IE | South America I | IE1 | IE1_2 |
| MF423377 | Carmo | 2017 | RJ | Alouatta | Brazil | IE | South America I | IE1 | IE1_1 |
| MF423378 | Carmo | 2017 | RJ | Alouatta | Brazil | IE | South America I | IE1 | IE1_1 |
| MF538782 | Sao Fidelis | 2017 | RJ | Human | Brazil | IE | South America I | IE1 | IE1_1 |
| MF538783 | Casimiro de Abreu | 2017 | RJ | Human | Brazil | IE | South America I | IE1 | IE1_2 |
| MF538784 | Porciuncula | 2017 | RJ | Human | Brazil | IE | South America I | IE1 | IE1_1 |
| MF434851 | Silva Jardim | 2017 | RJ | Human | Brazil | IE | South America I | IE1 | IE1_2 |
| MT497521 | Pindorama | 2016 | SP | Alouatta | Brazil | IE | South America I | IE2 | IE2_1 |
| MT497522 | Jaboticabal | 2016 | SP | Alouatta | Brazil | IE | South America I | IE2 | IE2_1 |
| MT497525 | Ribeirao Preto | 2017 | SP | Alouatta | Brazil | IE | South America I | IE2 | IE2_1 |
| MH030050 | Louveira | 2017 | SP | Callicebus | Brazil | IE | South America I | IE2 | IE2_3 |
| MH030049 | Vinhedo | 2017 | SP | Callithrix | Brazil | IE | South America I | IE2 | IE2_3 |

**Supplementary Table III (continued)**

| MH030053 | Itatiba | 2017 | SP | Alouatta | Brazil | IE | South America I | IE2 | IE2_3 |
| --- | --- | --- | --- | --- | --- | --- | --- | --- | --- |
| MH030051 | Louveira | 2017 | SP | Alouatta | Brazil | IE | South America I | IE2 | IE2_3 |
| MH030052 | Louveira | 2017 | SP | Alouatta | Brazil | IE | South America I | IE2 | IE2_3 |
| MH030055 | Jundiai | 2017 | SP | Alouatta | Brazil | IE | South America I | IE2 | IE2_3 |
| MH030056 | Jundiai | 2017 | SP | Alouatta | Brazil | IE | South America I | IE2 | IE2_3 |
| MH030054 | Jundiai | 2017 | SP | Alouatta | Brazil | IE | South America I | IE2 | IE2_3 |
| MH030062 | Itatiba | 2017 | SP | Alouatta | Brazil | IE | South America I | IE2 | IE2_3 |
| MH030059 | Itatiba | 2017 | SP | Alouatta | Brazil | IE | South America I | IE2 | IE2_3 |
| MH030061 | Itatiba | 2017 | SP | Alouatta | Brazil | IE | South America I | IE2 | IE2_3 |
| MH030058 | Itatiba | 2017 | SP | Alouatta | Brazil | IE | South America I | IE2 | IE2_2 |
| MH030057 | Braganca Paulista | 2017 | SP | Alouatta | Brazil | IE | South America I | IE2 | IE2_1 |
| MH030060 | Itatiba | 2017 | SP | Alouatta | Brazil | IE | South America I | IE2 | IE2_3 |
| MH030063 | Itatiba | 2017 | SP | Alouatta | Brazil | IE | South America I | IE2 | IE2_3 |
| MH030064 | Jundiai | 2017 | SP | Alouatta | Brazil | IE | South America I | IE2 | IE2_3 |
| MH030065 | Jundiai | 2017 | SP | Alouatta | Brazil | IE | South America I | IE2 | IE2_3 |
| MH030066 | Jundiai | 2017 | SP | Alouatta | Brazil | IE | South America I | IE2 | IE2_2 |
| MH030067 | Jundiai | 2017 | SP | Alouatta | Brazil | IE | South America I | IE2 | IE2_3 |
| MH030068 | Jundiai | 2017 | SP | Alouatta | Brazil | IE | South America I | IE2 | IE2_2 |
| MH030069 | São Paulo | 2017 | SP | Alouatta | Brazil | IE | South America I | IE2 | IE2_3 |
| MH030072 | Jarinu | 2017 | SP | Alouatta | Brazil | IE | South America I | IE2 | IE2_2 |
| MH030075 | Jarinu | 2017 | SP | Alouatta | Brazil | IE | South America I | IE2 | IE2_3 |
| MH030076 | Jarinu | 2017 | SP | Alouatta | Brazil | IE | South America I | IE2 | IE2_3 |
| MH030077 | Jarinu | 2017 | SP | Alouatta | Brazil | IE | South America I | IE2 | IE2_2 |
| MH030078 | Morungaba | 2017 | SP | Alouatta | Brazil | IE | South America I | IE2 | IE2_2 |
| MH030071 | Campo Limpo Paulista | 2017 | SP | Alouatta | Brazil | IE | South America I | IE2 | IE2_1 |
| MH030073 | Mairipora | 2017 | SP | Alouatta | Brazil | IE | South America I | IE2 | IE2_3 |
| MH030074 | Jarinu | 2017 | SP | Alouatta | Brazil | IE | South America I | IE2 | IE2_1 |
| MH030070 | Campo Limpo Paulista | 2017 | SP | Alouatta | Brazil | IE | South America I | IE2 | IE2_1 |

**Supplementary Table III (continued)**

| MH030079 | Nazare Paulista | 2017 | SP | Alouatta | Brazil | IE | South America I | IE2 | IE2_2 |
| --- | --- | --- | --- | --- | --- | --- | --- | --- | --- |
| MH030080 | Nazare Paulista | 2017 | SP | Alouatta | Brazil | IE | South America I | IE2 | IE2_1 |
| MH030081 | Campo Limpo | 2017 | SP | Alouatta | Brazil | IE | South America I | IE2 | IE2_2 |
| MH030082 | Campo Limpo | 2017 | SP | Alouatta | Brazil | IE | South America I | IE2 | IE2_2 |
| MH030083 | Campo Limpo | 2017 | SP | Alouatta | Brazil | IE | South America I | IE2 | IE2_3 |
| MH030084 | Piracaia | 2017 | SP | Alouatta | Brazil | IE | South America I | IE2 | IE2_3 |
| MH030085 | Piracaia | 2017 | SP | Alouatta | Brazil | IE | South America I | IE2 | IE2_3 |
| MH030086 | Piracaia | 2017 | SP | Alouatta | Brazil | IE | South America I | IE2 | IE2_2 |
| MH193173 | Guarulhos | 2018 | SP | Alouatta | Brazil | IE | South America I | IE2 | IE2_3 |
| MH193174 | Mairipora | 2018 | SP | Human | Brazil | IE | South America I | IE2 | IE2_2 |
| MH193175 | Sao Paulo | 2018 | SP | Alouatta | Brazil | IE | South America I | IE2 | IE2_3 |
| MZ604840 | Mairipora | 2018 | SP | Human | Brazil | IE | South America I | IE2 | IE2_1 |
| MZ604868 | Cotia | 2018 | SP | Human | Brazil | IE | South America I | IE2 | IE2_2 |
| MZ604842 | Mairipora | 2018 | SP | Human | Brazil | IE | South America I | IE2 | IE2_3 |
| MZ604845 | Serra da Cantareira | 2018 | SP | Human | Brazil | IE | South America I | IE2 | IE2_3 |
| MZ604870 | Horto Florestal | 2018 | SP | Human | Brazil | IE | South America I | IE2 | IE2_3 |
| MZ604856 | Guarulhos | 2018 | SP | Human | Brazil | IE | South America I | IE2 | IE2_2 |
| MZ604859 | Mairipora | 2018 | SP | Human | Brazil | IE | South America I | IE2 | IE2_3 |
| MZ604861 | Mairipora | 2018 | SP | Human | Brazil | IE | South America I | IE2 | IE2_1 |
| MZ604872 | Mairipora | 2018 | SP | Human | Brazil | IE | South America I | IE2 | IE2_3 |
| MZ604862 | Mairipora | 2018 | SP | Human | Brazil | IE | South America I | IE2 | IE2_3 |
| MZ604873 | Nazare paulista | 2018 | SP | Human | Brazil | IE | South America I | IE2 | IE2_1 |
| MZ604865 | Mairipora | 2018 | SP | Human | Brazil | IE | South America I | IE2 | IE2_2 |
| MZ604866 | Atibaia | 2018 | SP | Human | Brazil | IE | South America I | IE2 | IE2_1 |
| MZ604876 | Sao bernardo do campo | 2018 | SP | Human | Brazil | IE | South America I | IE2 | IE2_2 |
| MZ604838 | Pariquera_acu | 2019 | SP | Human | Brazil | IE | South America I | IE2 | IE2_4 |
| MZ604839 | Cananeia | 2019 | SP | Human | Brazil | IE | South America I | IE2 | IE2_4 |
| MZ604841 | Cajati | 2019 | SP | Human | Brazil | IE | South America I | IE2 | IE2_4 |

**Supplementary Table III (continued)**

| MZ604869 | Eldorado | 2019 | SP | Human | Brazil | IE | South America I | IE2 | IE2_4 |
| --- | --- | --- | --- | --- | --- | --- | --- | --- | --- |
| MZ604843 | Eldorado | 2019 | SP | Human | Brazil | IE | South America I | IE2 | IE2_4 |
| MZ604844 | Barra do Turvo | 2019 | SP | Human | Brazil | IE | South America I | IE2 | IE2_4 |
| MZ604846 | Cajati | 2019 | SP | Human | Brazil | IE | South America I | IE2 | IE2_4 |
| MZ604847 | Iporanga | 2019 | SP | Human | Brazil | IE | South America I | IE2 | IE2_4 |
| MZ604848 | Iporanga | 2019 | SP | Human | Brazil | IE | South America I | IE2 | IE2_3 |
| MZ604849 | Eldorado | 2019 | SP | Human | Brazil | IE | South America I | IE2 | IE2_3 |
| MZ604850 | Eldorado | 2019 | SP | Human | Brazil | IE | South America I | IE2 | IE2_3 |
| MZ604851 | Barra do Turvo | 2019 | SP | Human | Brazil | IE | South America I | IE2 | IE2_3 |
| MZ604852 | Iporanga | 2019 | SP | Human | Brazil | IE | South America I | IE2 | IE2_3 |
| MZ604853 | Barra do Turvo | 2019 | SP | Human | Brazil | IE | South America I | IE2 | IE2_4 |
| MZ604855 | Registro | 2019 | SP | Human | Brazil | IE | South America I | IE2 | IE2_3 |
| MZ604858 | Eldorado | 2019 | SP | Human | Brazil | IE | South America I | IE2 | IE2_3 |
| MZ604860 | Eldorado | 2019 | SP | Human | Brazil | IE | South America I | IE2 | IE2_3 |
| MZ604863 | Eldorado | 2019 | SP | Human | Brazil | IE | South America I | IE2 | IE2_4 |
| MZ604864 | Pariquera acu | 2019 | SP | Human | Brazil | IE | South America I | IE2 | IE2_4 |
| MZ604874 | Eldorado | 2019 | SP | Human | Brazil | IE | South America I | IE2 | IE2_3 |
| MZ604875 | Eldorado | 2019 | SP | Human | Brazil | IE | South America I | IE2 | IE2_4 |
| MZ604867 | Eldorado | 2019 | SP | Human | Brazil | IE | South America I | IE2 | IE2_3 |
| MZ604854 | Mairipora | 2019 | SP | Human | Brazil | IE | South America I | IE2 | IE2_4 |
| MZ604871 | Cananeia | 2019 | SP | Human | Brazil | IE | South America I | IE2 | IE2_4 |
| MZ604857 | Eldorado | 2019 | SP | Human | Brazil | IE | South America I | IE2 | IE2_4 |
| MN506291 | Valenca | 2018 | RJ | Haemagogus | Brazil | IE | South America I | IE1 | IE1_1 |
| MN506290 | Valenca | 2018 | RJ | Haemagogus | Brazil | IE | South America I | IE1 | IE1_1 |
| MN506289 | Valenca | 2018 | RJ | Haemagogus | Brazil | IE | South America I | IE1 | IE1_1 |
| MN506288 | Valenca | 2018 | RJ | Haemagogus | Brazil | IE | South America I | IE1 | IE1_1 |
| MN506287 | Valenca | 2018 | RJ | Haemagogus | Brazil | IE | South America I | IE1 | IE1_1 |
| MN506286 | Valenca | 2018 | RJ | Haemagogus | Brazil | IE | South America I | IE1 | IE1_1 |

**Supplementary Table III (continued)**

| MN506285 | Valenca | 2018 | RJ | Haemagogus | Brazil | IE | South America I | IE1 | IE1_1 |
| --- | --- | --- | --- | --- | --- | --- | --- | --- | --- |
| MN506284 | Valenca | 2018 | RJ | Haemagogus | Brazil | IE | South America I | IE1 | IE1_1 |
| MN506283 | Marica | 2017 | RJ | Haemagogus | Brazil | IE | South America I | IE1 | IE1_2 |
| MN506282 | Marica | 2017 | RJ | Haemagogus | Brazil | IE | South America I | IE1 | IE1_2 |
| MN506281 | Macae | 2017 | RJ | Haemagogus | Brazil | IE | South America I | IE1 | IE1_2 |
| MN506280 | Marica | 2017 | RJ | Haemagogus | Brazil | IE | South America I | IE1 | IE1_2 |
| MN506279 | Marica | 2017 | RJ | Haemagogus | Brazil | IE | South America I | IE1 | IE1_2 |
| MN506278 | Marica | 2017 | RJ | Haemagogus | Brazil | IE | South America I | IE1 | IE1_2 |
| MN506277 | Marica | 2017 | RJ | Haemagogus | Brazil | IE | South America I | IE1 | IE1_2 |
| MN506276 | Marica | 2017 | RJ | Haemagogus | Brazil | IE | South America I | IE1 | IE1_2 |
| MN506275 | Marica | 2017 | RJ | Haemagogus | Brazil | IE | South America I | IE1 | IE1_2 |
| MN506274 | Marica | 2017 | RJ | Aedes | Brazil | IE | South America I | IE1 | IE1_2 |
| MN506273 | Marica | 2017 | RJ | Haemagogus | Brazil | IE | South America I | IE1 | IE1_2 |
| MN506272 | Marica | 2017 | RJ | Aedes | Brazil | IE | South America I | IE1 | IE1_2 |
| MN506271 | Marica | 2017 | RJ | Haemagogus | Brazil | IE | South America I | IE1 | IE1_2 |
| MN506270 | Marica | 2017 | RJ | Haemagogus | Brazil | IE | South America I | IE1 | IE1_2 |
| MN506269 | Marica | 2017 | RJ | Haemagogus | Brazil | IE | South America I | IE1 | IE1_2 |
| MN506268 | Marica | 2017 | RJ | Haemagogus | Brazil | IE | South America I | IE1 | IE1_2 |
| MN506267 | Marica | 2017 | RJ | Haemagogus | Brazil | IE | South America I | IE1 | IE1_2 |
| MN506266 | Nova Iguacu | 2018 | RJ | Haemagogus | Brazil | IE | South America I | IE1 | IE1_2 |
| MN506265 | Teresopolis | 2017 | RJ | Haemagogus | Brazil | IE | South America I | IE1 | IE1_1 |
| JF912179 | Uruacu | 1980 | GO | Haemagogus | Brazil | IC | South America I | IC | IC |
| JF912180 | C do Araguaia | 1981 | PA | Human | Brazil | - | South America I | - | - |
| JF912181 | Porto_Velho | 1983 | RO | Human | Brazil | II | South America II | II | II |
| JF912182 | Monte_Alegre | 1984 | PA | Human | Brazil | IB | South America I | IB | IB |
| JF912183 | Sao Domingos do Capim | 1984 | PA | Human | Brazil | IC | South America I | IC | IC |
| JF912184 | Breves | 1987 | PA | Human | Brazil | IC | South America I | IC | IC |
| JF912185 | Sidrolandia | 1992 | MS | Sabethes | Brazil | IB | South America I | IB | IB |

**Supplementary Table III (continued)**

| JF912186 | Arinos | 1994 | MG | Human | Brazil | IC | South America I | IC | IC |
| --- | --- | --- | --- | --- | --- | --- | --- | --- | --- |
| JF912187 | Goias | 2000 | GO | Human | Brazil | ID | South America I | ID | ID |
| JF912188 | Alto_Paraiso | 2000 | GO | Human | Brazil | ID | South America I | ID | ID |
| JF912189 | Missoes | 2001 | RS | Haemagogus | Brazil | ID | South America I | ID | ID |
| JF912190 | Alto Alegre | 2002 | RR | Human | Brazil | IE | South America I | IE | IE |

**Supplementary Table IV**

**Single nucleotide variations detected by NGS. Non-synonymous variants areindicated. Frequencies and correspondent outbreaks are listed below.** *nt:nucleotide; (-) without information; NGS: new generation sequencing; A: alanine; D: aspartic acid; G: glycine; H: Histidine; I: isoleucine; K: lysine; L: leucine; M: methionine; N: asparagine; P: proline; R: arginine; T: threonine; V: valine; W: tryptophan

| **Reference(nt)** | **Protein** | **Reference** | **Allelevariation** | **Outbreakyeak** | **Frequency** | **Aminoacid change** |
| --- | --- | --- | --- | --- | --- | --- |

| 195 | C | A | A/G | 2019 | (15/39) | K26R |
| --- | --- | --- | --- | --- | --- | --- |
| 218 | C | A | A/G | 2018 | (4/39) | I34V |
| 297 | C | A | A/G | 2019 | (1/39) | K60R |
| 1021 | E | C | C/T | 2019 | (1/39) | H301Y |
| 1140 | E | C | C/T | 2019 | (1/39) | A341V |
| 1781 | E | A | A/G | 2019 | (1/39) | N555D |
| 1861 | E | C | C/T | 2018/2019 | (36/39) | - |
| 1908 | E | A | A/G | 2018 | (1/39) | D597G |
| 2975 | NS1 | T | T/C | 2018 | (1/39) | Y953H |
| 3097 | NS1 | T | T/C | 2018/2019 | (39/39) | - |
| 3099 | NS1 | T | T/C | 2019 | (1/39) | L994P |
| 3317 | NS1 | G | G/A | 2019 | (1/39) | G1067R |
| 3502 | NS1 | A | A/G | 2018/2019 | (39/39) | - |
| 3744 | NS2A | C | C/T | 2019 | (1/39) | A1209V |
| 4948 | NS3 | T | T/C | 2018/2019 | (39/39) | - |
| 4966 | NS3 | C | C/T | 2018/2019 | (39/39) | - |
| 5055 | NS3 | A | A/C | 2018/2019 | (39/39) | T1646I |
| 5231 | NS3 | C | C/T | 2018/2019 | (39/39) | - |
| 5290 | NS3 | T | T/C | 2019 | (24/39) | - |
| 5335 | NS3 | T | T/C | 2018/2019 | (39/39) | - |
| 5362 | NS3 | C | C/T | 2018/2019 | (39/39) | - |
| 5452 | NS3 | G | G/A | 2018/2019 | (39/39) | - |
| 5533 | NS3 | T | T/C | 2018/2019 | (39/39) | - |
| 5595 | NS3 | C | C/T | 2018 | (1/39) | T1826M |
| 5839 | NS3 | C | C/T | 2018/2019 | (39/39) | - |

**Supplementary Table IV (continued)**

**Single nucleotide variations detected by NGS. Non-synonymous variants areindicated. Frequencies and correspondent outbreaks are listed below.** *nt:nucleotide; (-) without information; NGS: new generation sequencing; A: alanine; D: aspartic acid; G: glycine; H: Histidine; I: isoleucine; K: lysine; L: leucine; M: methionine; N: asparagine; P: proline; R: arginine; T: threonine; V: valine; W: tryptophan

| **Reference(nt)** | **Protein** | **Reference** | **Allelevariation** | **Outbreakyeak** | **Frequency** | **Aminoacid change** |
| --- | --- | --- | --- | --- | --- | --- |

| 5976 | NS3 | C | C/A | 2018/2019 | (2/39) | P1953H |
| --- | --- | --- | --- | --- | --- | --- |
| 5989 | NS3 | G | G/A | 2018/2019 | (39/39) | - |
| 6199 | NS3 | T | T/C | 2018/2019 | (39/39) | - |
| 6525 | NS4A | T | T/G | 2018/2019 | (5/39) | V2136G |
| 6526 | NS4A | GCTC | GCTC/TCTT | 2019 | (24/39) | - |
| 6528 | NS4A | T | T/C | 2018 | (1/39) | L2137P |
| 6586 | NS4A | G | G/A | 2018/2019 | (39/39) | - |
| 6623 | NS4A | C | C/T | 2019 | (24/39) | - |
| 6934 | NS4B | G | G/A | 2018/2019 | (39/39) | - |
| 7231 | NS4B | C | C/T | 2018/2019 | (39/39) | - |
| 7240 | NS4B | A | A/T | 2018/2019 | (39/39) | - |
| 7721 | NS5 | C | C/T | 2018 | (1/39) | R2535W |
| 7976 | NS5 | A | A/G | 2019 | (1/39) | M2620V |
| 8542 | NS5 | G | G/A | 2018/2019 | (39/39) | - |
| 8569 | NS5 | C | C/A | 2018/2019 | (39/39) | - |
| 8638 | NS5 | C | C/T | 2018/2019 | (39/39) | - |
| 8746 | NS5 | G | G/A | 2019 | (24/39) | - |
| 9564 | NS5 | C | C/T | 2018 | (1/39) | A3149V |
| 9775 | NS5 | C | C/T | 2018/2019 | (39/39) | - |
| 9982 | NS5 | T | T/C | 2018/2019 | (39/39) | - |
| 10104 | NS5 | C | C/T | 2018/2019 | (30/39) | T3329I |

**References**

1. Faria NR, Kraemer MUG, Hill SC, Goes de Jesus J, Aguiar RS, Iani FCM, et al.Genomicandepidemiologicalmonitoringofyellowfevervirustransmissionpotential.Science.2018;361(6405):894-9.
