# Supplementary figures and images for: Updating the Phylodynamics of Yellow Fever Virus 2016–2019 Brazilian Outbreak With New 2018 and 2019 São Paulo Genomes
Source: Front Microbiol. 2022 Apr 14;13:811318. doi: 10.3389/fmicb.2022.811318 (PMC9132216; doi:10.3389/fmicb.2022.811318)

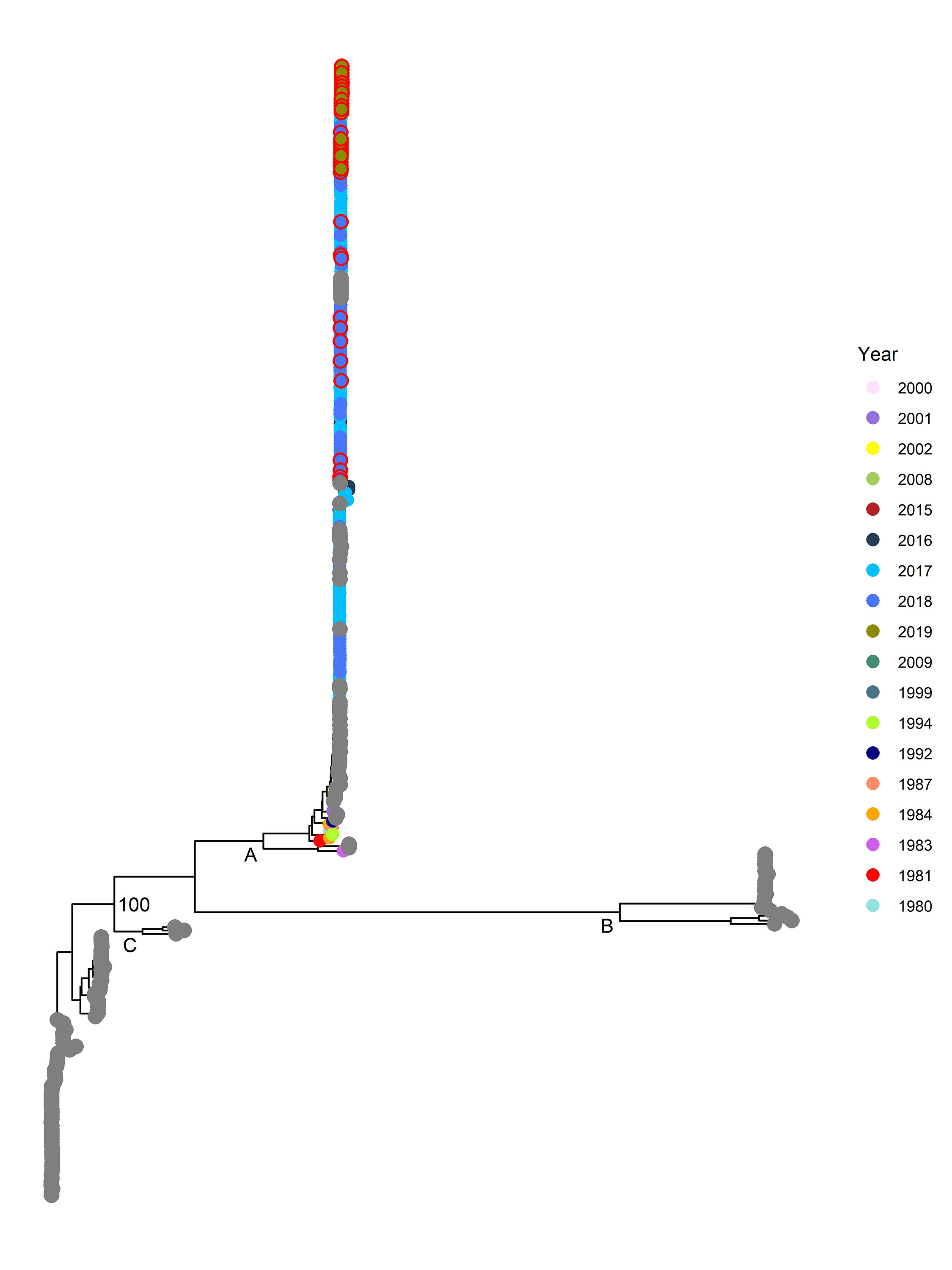

Supplement: Supplementary file 3 [file Image_1.JPEG]

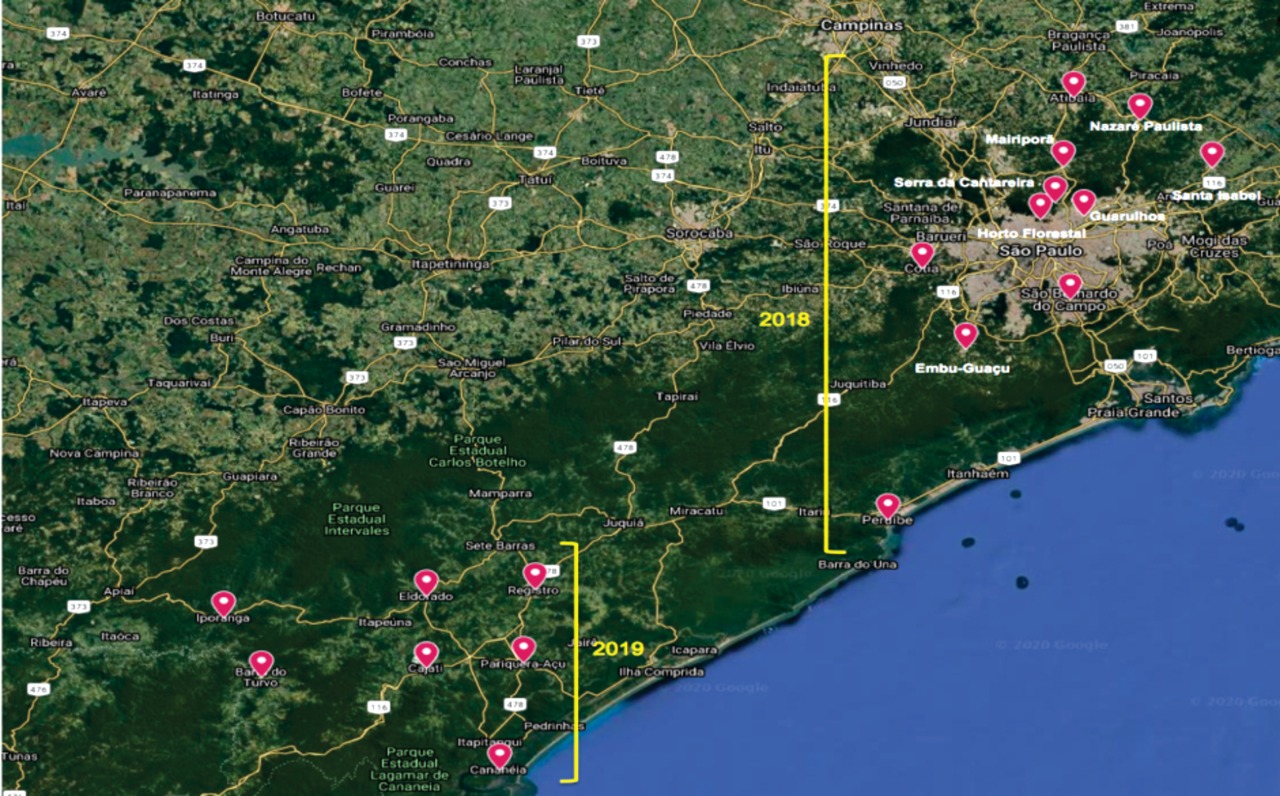

Supplement: Supplementary file 4 [file Image_2.JPEG]

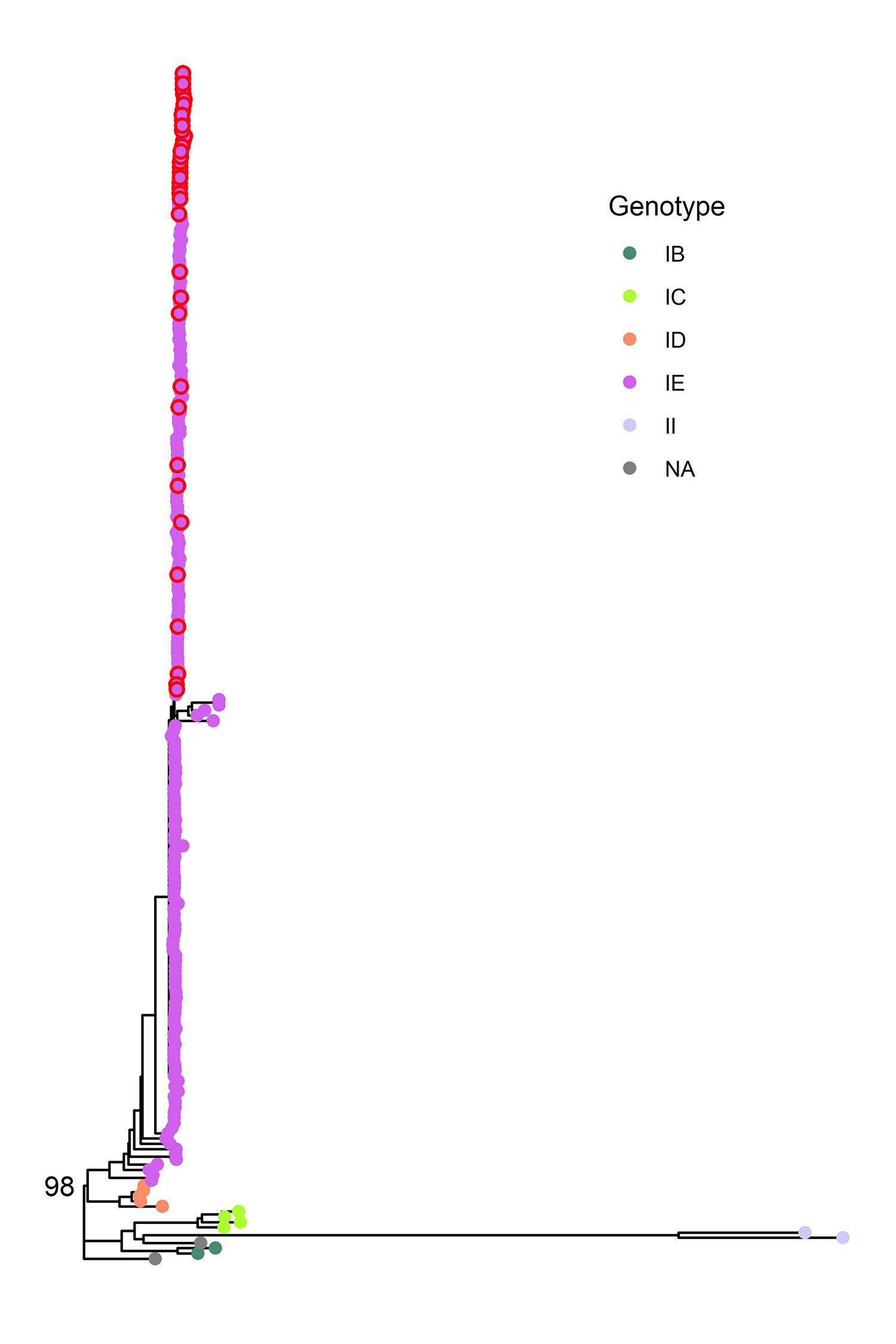

Supplement: Supplementary file 5 [file Image_3.JPEG]

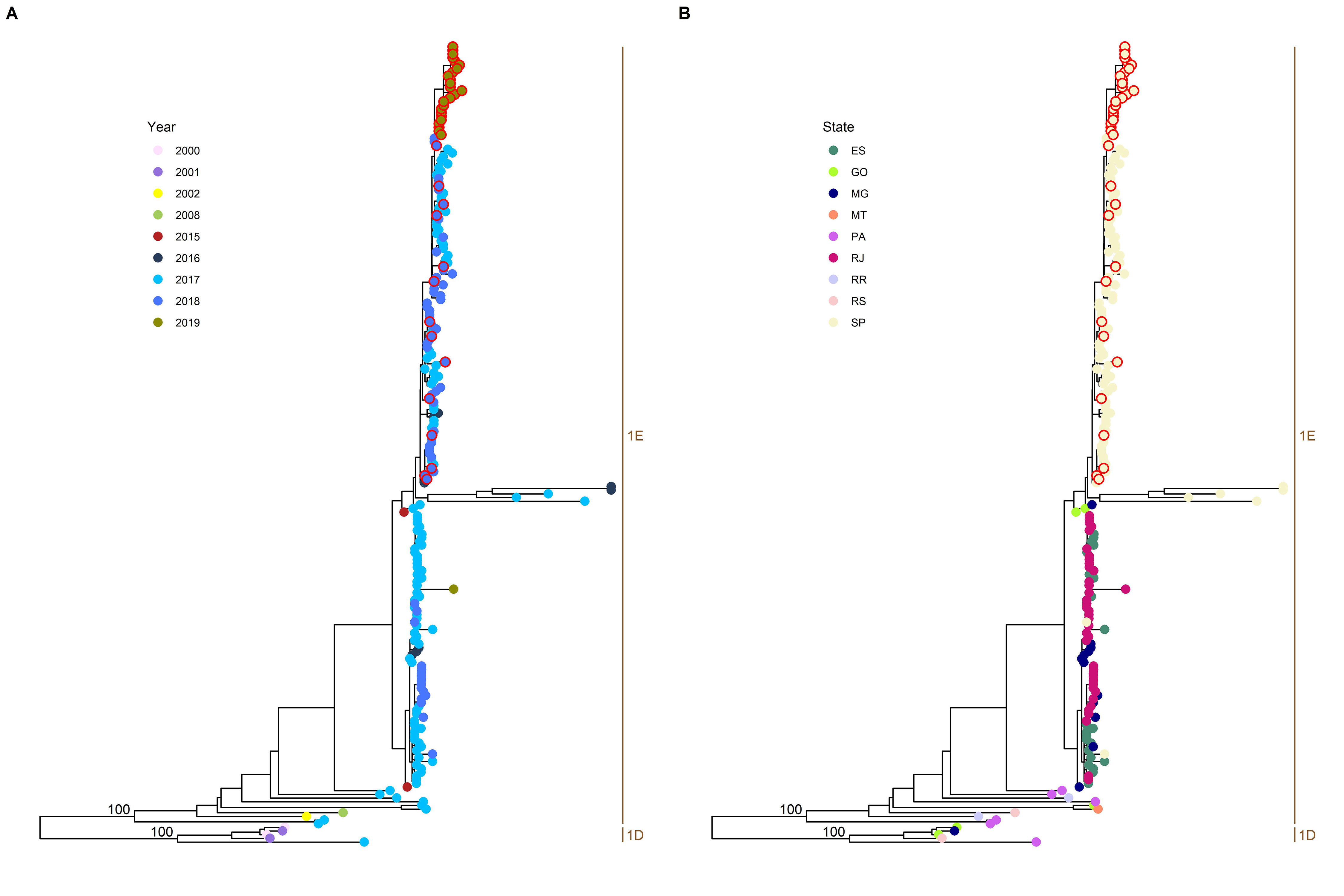

Supplement: Supplementary file 6 [file Image_4.JPEG]

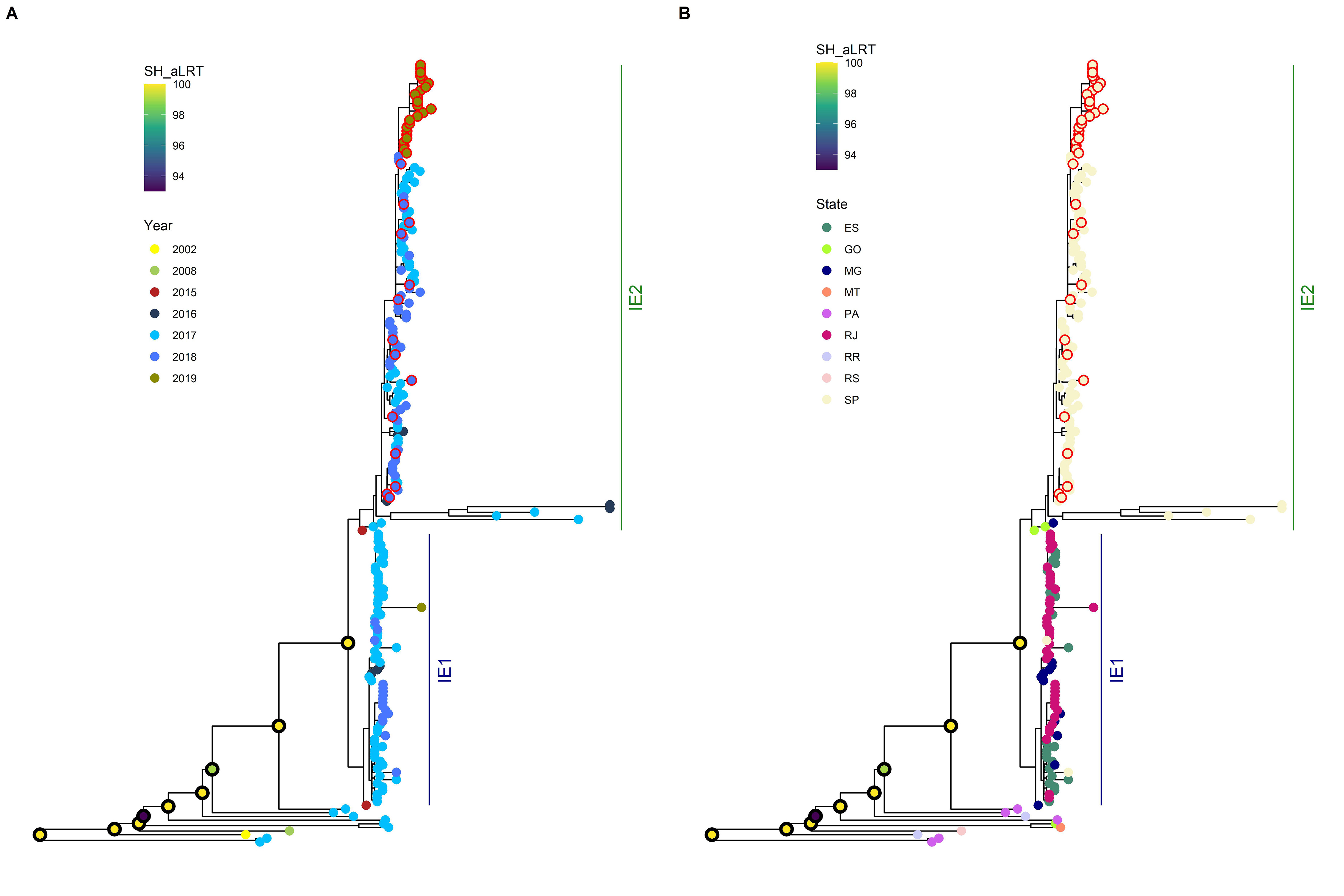

Supplement: Supplementary file 7 [file Image_5.JPEG]

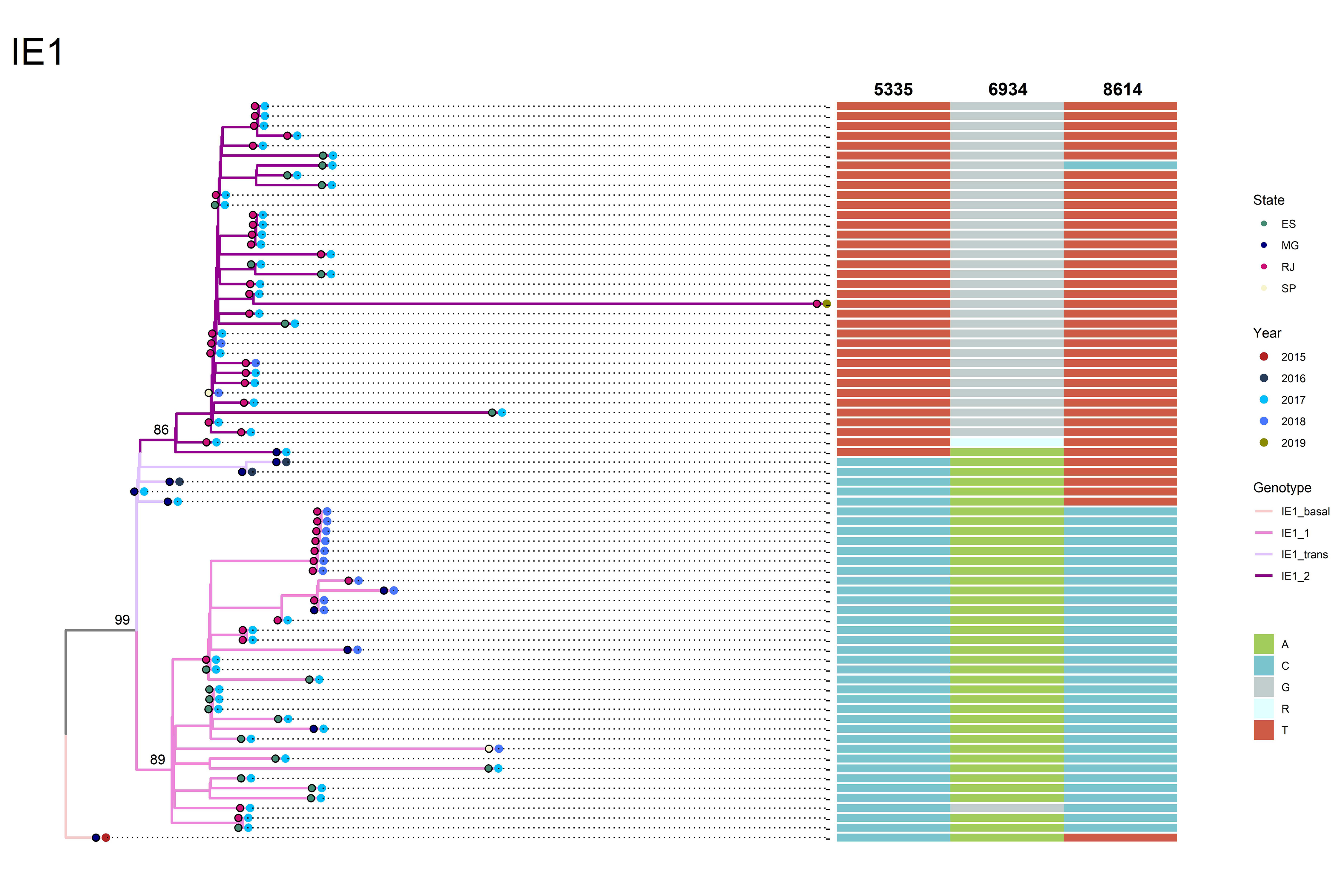

Supplement: Supplementary file 8 [file Image_6.JPEG]

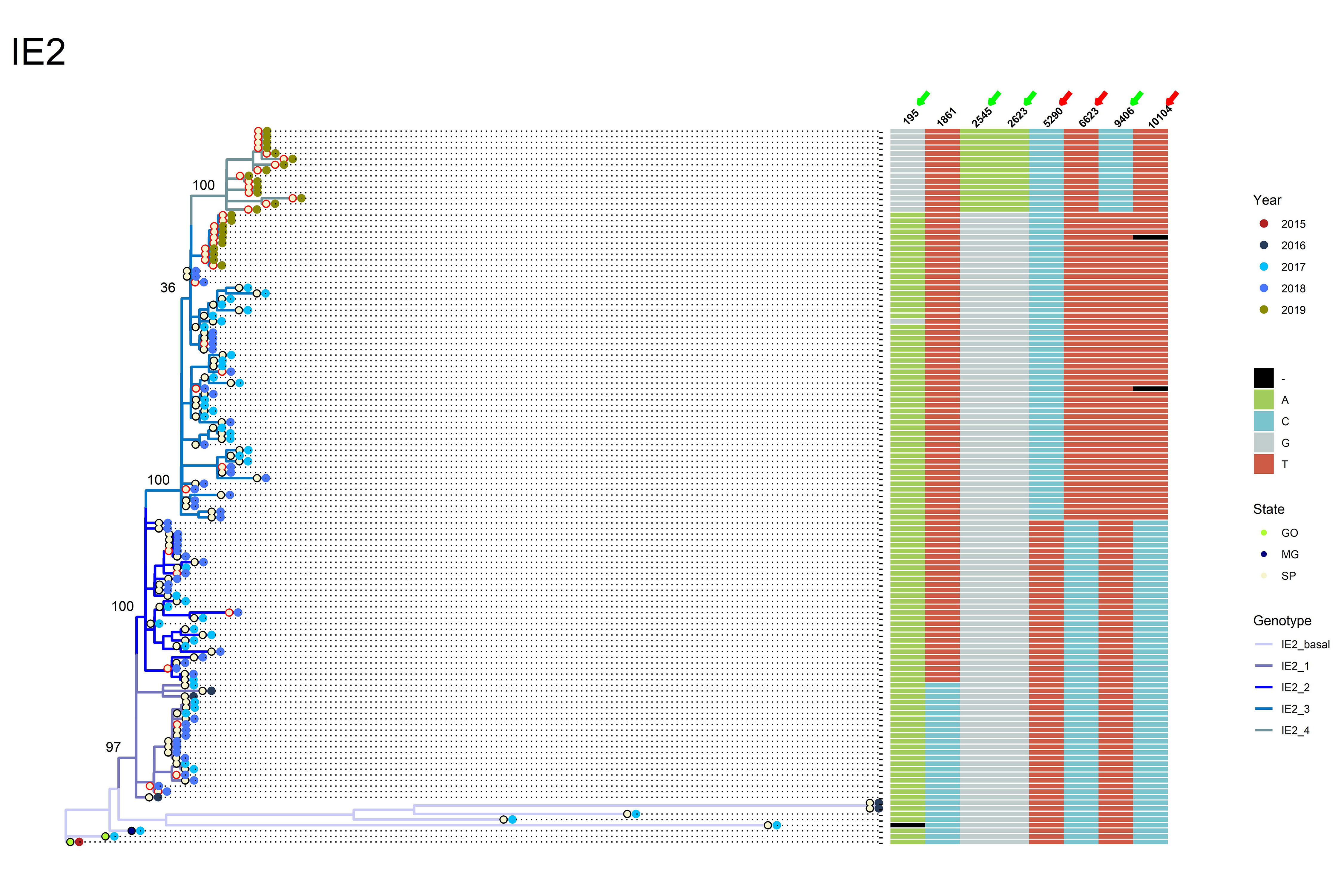

Supplement: Supplementary file 9 [file Image_7.JPEG]

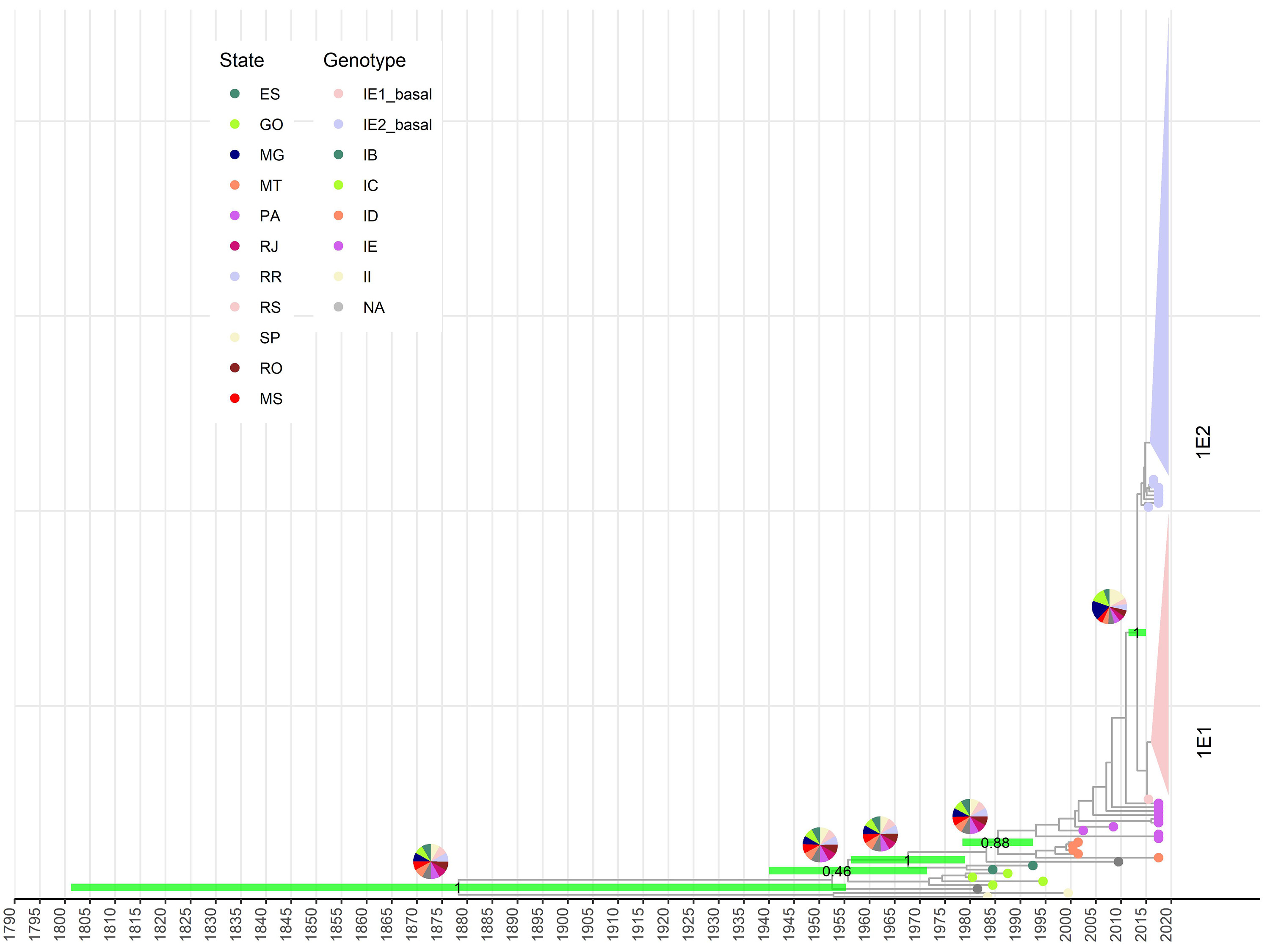

Supplement: Supplementary file 10 [file Image_8.JPEG]
